# Polymer Delivery Systems for Long-Acting Antiretroviral Drugs

**DOI:** 10.3390/pharmaceutics16020183

**Published:** 2024-01-28

**Authors:** Mohammad Ullah Nayan, Sudipta Panja, Ashrafi Sultana, Lubaba A. Zaman, Lalitkumar K. Vora, Brady Sillman, Howard E. Gendelman, Benson Edagwa

**Affiliations:** 1 Department of Pharmacology and Experimental Neuroscience, University of Nebraska Medical Center, Omaha, NE 68198-5880, USA; mohammadullah.nayan@unmc.edu (M.U.N.); sudipta.panja@unmc.edu (S.P.); asultana@unmc.edu (A.S.); lubaba.zaman@unmc.edu (L.A.Z.); brady.sillman@unmc.edu (B.S.); 2 School of Pharmacy, Queen’s University Belfast, Medical Biology Centre, Belfast BT9 7BL, UK; l.vora@qub.ac.uk

**Keywords:** long-acting formulations, polymer, antiretroviral therapy, HIV, chronic infectious diseases, implants, vaginal rings, prodrug nanoformulations, microarray patches

## Abstract

The success of long-acting (LA) drug delivery systems (DDSs) is linked to their biocompatible polymers. These are used for extended therapeutic release. For treatment or prevention of human immune deficiency virus type one (HIV-1) infection, LA DDSs hold promise for improved regimen adherence and reduced toxicities. Current examples include Cabenuva, Apretude, and Sunlenca. Each is safe and effective. Alternative promising DDSs include implants, prodrugs, vaginal rings, and microarray patches. Each can further meet patients’ needs. We posit that the physicochemical properties of the formulation chemical design can optimize drug release profiles. We posit that the strategic design of LA DDS polymers will further improve controlled drug release to simplify dosing schedules and improve regimen adherence.

## 1. Introduction

Long-acting (LA) drug delivery systems (DDSs) harness polymer properties to achieve spatiotemporal control over release and drug biodistribution. This allows the LA dosage regimens to extend from days to months. Such DDSs significantly reduce the burden of chronic disease. Treatment success parallels medication adherence. The development of LA medicines is traced back to the 1930s with the discovery of hydrophobic drug release from implants [1]. However, the discovery of low-molecular-weight (MW) dye diffusion through silicone tubing laid the groundwork for rate-controlling LA DDS polymers [2]. Biopharmaceutical research activities directed their efforts to convert biocompatible silicone tubing into versatile drug delivery materials. These efforts included improved uses of atropine, histamine, anesthetic, steroid, antimalarial, and antischistosomal agents [2]. Since the 1960s, the work of Drs. Folkman, Langer, Higuchi, Roseman, Peppas, Heller, Ringsdorf, and Speiser have pioneered the LA DDSs advancements seen today. Widespread research resulted in the development of LA DDSs which extended the dosage intervals for a broad range of drug regimens. This resulted in improved treatment outcomes for contraception and for treatments of psychosis, diabetes, osteoporosis, and ocular diseases. 

A key example of a treatable chronic disease that would benefit from LA DDSs is human immune deficiency virus type one (HIV-1) infection. Here, prior studies demonstrated that non-adherence to therapeutic regimens was a significant limiting factor in achieving successful treatment outcomes. One notable point is the development of viral resistance. Another is simply remembering to take the medicines according to daily prescribed requirements. Indeed, patients have repeatedly expressed treatment preferences for infrequent dosing regimens as, without a viable cure or preventive vaccine, daily oral antiretroviral therapy (ART) is a principal means for HIV-1 treatment and prevention. Over the past two decades, considerable efforts have been focused on the development of LA ARTs that include a specific focus on controlled-release LA formulations. Such formulations include solid implants, vaginal rings (VRs), and surfactant-stabilized aqueous nanocrystal suspensions. In these depot-forming DDSs, the drug is either encapsulated in biodegradable polymers for implantation in subcutaneous (SC) space or in reproductive organs or formulated as drug nanocrystals for either intramuscular (IM) or SC injections. Extensive tests have culminated in the approval of surfactant-stabilized aqueous nanocrystal LA cabotegravir and rilpivirine (CAB and RPV LA) suspensions by the US Food and Drug Administration (FDA). CAB and RPV LA offer the convenience of monthly or bimonthly dosing for HIV-1 treatment [3,4,5].

Multiple randomized clinical trials have demonstrated comparable efficacy between CAB and RPV LA and a standard daily oral regimen [4,6,7]. Based on the data that 91% of prior clinical trial participants preferred LA ART over daily oral medicines, CAB LA proved more effective in preventing HIV-1 infection when compared to daily oral Truvada [8]. This propelled its US FDA approval in December 2021 [5]. The remarkable efficacy of CAB LA was linked to improved treatment satisfaction and therapeutic adherence. However, the limitations associated with CAB LA include the need for frequent clinical visits, injection site reactions, variable pharmacokinetics (PK) profiles, prolonged terminal phase tailings, high costs, and comorbid conditions.

Alternative approaches that include in-situ-forming implants (ISFIs), solid implants (SIs), and prodrug nanocrystals are being developed to further improve the PK profiles and other listed limitations associated with CAB LA. Notably, surfactant-coated stearoylated CAB prodrug nanocrystals achieved sustained therapeutic drug levels for up to a year in preclinical models [9]. Other studies demonstrated that a poly(lactic acid-co-glycolic acid) (PLGA)-based ISFI could potentially extend CAB dosing intervals to every 6 months [10]. Other studies have also shown that solid implants containing highly potent ARVs, such as tenofovir alafenamide (TAF) and islatravir (ISL), can simplify ART dosages [11,12,13,14]. However, in November 2021, Merck announced that participants who received once-weekly ISL plus MK-8507 experienced a decline in CD4 T-cells. Further review found that CD4 counts also fell in people taking once-daily ISL plus doravirine for HIV-1 treatment, while those taking ISL alone for pre-exposure prophylaxis (PrEP) experienced a decline in total lymphocyte counts. As a result, studies of ISL for PrEP have been discontinued. These findings underscore the importance of establishing safety profiles of new chemical entities prior to combining them with other therapies. Polymeric TAF implant technologies have demonstrated the zero-order release kinetics of the pharmacologically active tenofovir diphosphate (TFV-DP) metabolite in preclinical models of PK studies [15]. These include non-degradable materials that provide sustained drug delivery. The use of self-administered microbicides can significantly reduce the risk of HIV-1 infection. Vaginal rings (VRs) that consist of non-biodegradable elastomeric polymers are being developed as self-administrable PrEP treatment targeting at-risk populations [16]. Several VRs of TAF and dapivirine (DPV), a non-nucleoside reverse transcriptase inhibitor (NNRTI), have demonstrated that such formulations could extend ART dosing intervals [16,17,18]. In two separate phase III clinical trials, known as the RING and the ASPIRE studies, DPV VRs demonstrated their effectiveness in reducing HIV-1 transmission. Compared to a placebo ring, the DPV ring reduced transmission rates by 31% in the RING study and 27% in the ASPIRE study [19,20]. These results suggest that the DPV ring could supplement existing HIV prevention practices. Microneedles (MNs), also referred to as microarray patches (MAPs), are another class of LA DDS that use drug-diffusible, rate-controlling, non-biodegradable, or slowly biodegradable polymer membranes to extend the apparent half-lives of drugs [21]. Due to the non-invasive and self-administrable nature, the goal of MNs is to expand LA ART options and to appeal to a wide variety of users that include pediatric populations. Examples include CAB- and RPV-loaded MNs that are currently being explored as alternative treatment options to daily oral therapy as well as injectables [22,23]. However, further work is required to demonstrate improved or comparable PK and efficacy profiles to the existing CAB and RPV LA injectable formulations.

This review summarizes the physiochemical and biological parameters of the commonly used polymers used for LA DDSs in treatment and prevention regimens for HIV-1 infection. LA ART formulations either in preclinical or clinical development are discussed. For each of these delivery systems, polymer compositions and drug release kinetics are discussed. Additionally, an expert opinion section highlights LA ART DDS design considerations which could potentially encourage widespread acceptance and utilization among the key target populations.

## 2. LA DDSs

The emergence of polymer-based DDSs can be traced back to the 1930s when implanted pellets with hydrophobic substances were identified for their ability to facilitate sustained drug release [24]. Examples include pellets with estradiol for prostate cancer treatment and testosterone pellets for the treatment of hypogonadism [25]. Later, clinical use of depot-forming formulations loaded with hydrophobic drugs in either water-based or oily mediums, such as procaine penicillin G in water and fluphenazine decanoate in sesame oil, became popular [26,27]. Since then, more drug delivery approaches and models have been developed to improve our understanding of the materials and drug release mechanisms. In the 1960s, T. Higuchi presented his renowned ‘Higuchi model’ to describe the drug release kinetics from various sustained release matrix systems [28]. The model suggested that the release of the solid drugs dispersed in a matrix varies with the square root of time. At the same time, Folkman discovered that silicone rubber could act as a drug reservoir, allowing for constant drug release after implantation. This breakthrough led to the conceptualization of rate-controlling membranes or reservoir-based implants [29]. Notable examples of this concept in action are Ocuserts^®^, which are designed to deliver ocular drugs at predictable and controlled rates [30].

The challenges associated with non-biodegradable implants, such as the necessity of surgical removal after product life ends and adverse implant site reactions, spurred the development of biodegradable implants. The development and clinical utilization of biodegradable polymers like polylactic acid (PLA), polyglycolide (PGA), and PLGA date back to the 1970s [31]. At the early stage of development, clinical applications of these biodegradable polymers were restricted to surgical sutures [31]. Later on, PLA and PLGA microparticles and pellet depot systems for delivering contraceptive drugs and luteinizing-hormone-releasing hormone (LHRH) analogs were developed [32]. Other notable examples of commercial products where the biodegradable copolymers were utilized include Lupron Depot and Zoladex^®^ implants for treatment of prostate cancer, breast cancer, and endometriosis-like disease [32]. Furthermore, a polymer-based VR was patented by Upjohn Company for sustained drug release [33]. These developments marked significant milestones in the evolution of polymer-based LA DDSs. Additionally, extensive research was undertaken to develop silicone-based VRs for contraception. In-depth research, primarily sponsored by the World Health Organization (WHO), paved the way for the clinical approval of multiple contraceptive implants in the early 1990s.

**Figure 1:**
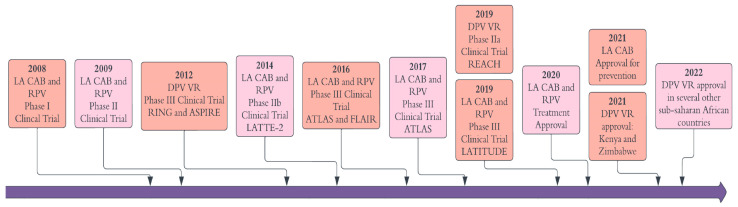
Timeline for LA drug delivery formulations for the treatment and prevention of HIV-1.

The development of LA ARTs began in the early 2000s, with an initial focus on VRs and implants. Later, attention shifted towards nanocrystal formulations [34]. Clinical trials for nanocrystal aqueous suspensions started around 2008 (Figure 1), including phase II trials for HIV-1 prevention (PrEP) with LA RPV [35]. However, the development of this formulation as a single agent for PrEP faced challenges linked to the high prevalence of NNRTI mutations [35,36]. After a decade of research and several clinical trials, the first LA complete therapy for HIV-1 treatment was approved in 2020 [37].

## 3. Drug Release Kinetics from LA DDSs

In polymer-based DDSs, drug release refers to a transfer process in which drug molecules are released from the inner core or matrix to the outer surface of the delivery system and eventually into the surrounding environment or tissue [38]. The rate of drug release from polymer-based DDSs can be modulated by choosing an appropriate polymer with a suitable DDS design. The terminology ‘long-acting’ refers to the ability to extend the duration of action of a therapeutic agent for a longer period of time. Most often, the terms long-acting, controlled release, sustained release, and extended release are used synonymously [39]. According to the United States Pharmacopeia (USP), the term ‘extended release’ or ‘long-acting’ or ‘sustained release’ is defined as ‘a deliberate modification to protract the release rate of an active pharmaceutical ingredient (API) in comparison to an immediate release dosage form’ [40]. In this review, the terminology ‘long-acting’, ‘sustained release’, or ’controlled release’ is used to designate formulations that can extend the apparent half-lives of drugs. The release of drugs from a delivery system can have one or more mechanisms, which can be correlated with a number of existing release kinetics models. The commonly used models are zero-order kinetic, first-order kinetic, Higuchi, Korsmeyer–Peppas, Peppas–Sahlin, and Hixson–Crowell. The zero-order kinetic model considers that there is no drug concentration influence on drug release rates. The zero-order kinetic models are reflected by the mathematical Equation (1), where C_0_ and C_t_ represent the amount of drug at the start and the amount released at time ‘t’.

(1) $$C_{0}-C_{t}=K_{0}t$$

For first-order kinetics, the rate of drug release is proportional to the concentration of the remaining drug in the delivery system and represented by Equation (2), where dc/dt is the rate of drug release, K_1_ is the first-order rate constant, and C_t_ is the concentration of the remaining drug at time t.

(2) $$\frac{dC}{dt}=-K_{1}C_{t}$$

The Korsmeyer–Peppas model (Equation (3)) reflects dissolution-mediated drug release. ‘M_t_’ and ‘M_∞_’ are the amount of eluted drug, ‘t’ is the recorded time ‘∞’.The ratio of ‘M_t_/M_∞_’ denotes the fraction of drug release at the time ‘t’.K_k_ is the Korsmeyer rate constant.

(3) $$\frac{M_{t}}{M_{\infty }}=Kt^{n}$$

The Peppas–Sahlin equation (Equation (4)) links to the diffusion- and relaxation-mediated drug release. The diffusion exponent is represented by ‘m’, and kinetic constants are represented by K_1_ and K_2_.

(4) $$\frac{M_{t}}{M_{\infty }}=K_{1}t^{m}+K_{2}t^{2m}$$

The Higuchi model (Equation (5)) is based on diffusion-mediated drug release. Here, ‘Q’ indicates the amount of drug released per unit area at time t and K_H_ is the Higuchi constant.

(5) $$Q=K_{H}\sqrt{t}$$

The Hixson–Crowell model (Equation (6)) applies to uniform-size drug particles, where the rate of drug release is controlled by drug dissolution. This is governed by the surface area or diameter of the drug-encased particles. The rate of drug dissolution is based on the cube root of the drug mass. The M_0_ and M_t_ are the mass of the drug at the initial and at the recorded time ‘t’. K_HC_ symbolizes the Hixson–Crowell release constant.

(6) $$M_{0}^{1/3}-M_{t}^{1/3}=K_{HC}t$$

An ideal LA DDS should exhibit zero-order drug release kinetics where a constant amount of drug is released per unit time. However, maintaining zero-order kinetics is very challenging, and it majorly depends on the physicochemical properties of the drug and excipients [41,42]. Diffusion, osmotic pumping, swelling, degradation, or erosion-induced release are the commonly reported mechanisms for polymer-based DDSs.

Non-degradable polymer-based DDSs can be either reservoir or matrix form. In the reservoir form of DDSs, the release rate is governed by the thickness of the polymer membrane and permeability of the drug through the polymer membrane. Whereas, in the matrix form of DDSs, Fickian diffusion remains the underlying release mechanism [43]. Diffusion refers to the random movement of the drug molecules from the higher- to the lower-concentration region in the matrix. The rate of diffusion in LA DDSs can be described by Fick’s law [43]. According to this law, the drug release rate depends on the concentration gradient (ΔC) and diffusibility (D) of the drug through the polymer matrix (Equation (7)). Particularly for slab-like matrix, Equation (7) can be simply transformed to Equation (8).

(7) $$\frac{\partial C}{\partial t}=D\Delta C\;$$

D is the diffusion coefficient or diffusivity, C is the concentration.

(8) $$\frac{M_{t}}{M_{0}}=4{\left(\frac{Dt}{\pi h^{2}}\right)}^{2}$$

M_t_ is the sum of drug released at time t, M_0_ is the total of the drug-loaded mass, D is the diffusion coefficient, and h is the thickness of the slab-like matrix.

According to Equation (8), the release rate is directly proportional to the drug’s diffusibility (D) through the slab and inversely proportional to the thickness (h) of the polymer [43]. Although Equation (8) can be transformed to other various forms based on the geometry of the DDSs, the parameters, such as the drug’s diffusibility and slab thickness, play their role in a similar way. For a particular DDS, the release kinetics can be zero order when ‘h’ and ‘D’ remain constant over time (Equation (8)) [43]. The diffusibility is influenced by the size of the drug molecule relative to the pore size of the matrix. Furthermore, matrix pore size and density have been governed by the properties of the polymer used to fabricate the matrix, such as nature of the monomers and the molar composition used to synthesize the polymer [44]. Matrix systems lack a rate-controlling membrane, so the diffusion rate is affected by non-constant drug concentration gradient and diffusion distance. Additionally, the diffusion distance is reliant on the polymer’s swelling [44]. 

In degradable polymer-based DDSs, drug release is majorly controlled by the rate of polymer degradation or erosion and osmotic pumping of the drugs. The chemical degradation of the polymer is influenced by its hydrophilicity. Hydrophilic polymers can absorb water, resulting in an increase in their pore size. This allows them to initiate drug release [45]. Over time, the polymer undergoes degradation, which increases the number and size of pores, eventually leading to continuous release of drug. Unlike degradation, erosion is an alternative method of the drug release process where polymeric chain segments are dissolved by keeping their chemical structure intact [44]. Erosion processes can happen either on the surface or bulk or a combination of both places on the DDS. Surface erosion gradually reduces the size of the DDS from the outside in [46,47]. In bulk erosion, water permeates the entire bulk of the polymer matrix, leading to a uniform degradation with no significant change in their initial size [47]. In addition, bulk erosion may produce faster and unpredictable drug release kinetics, making it less favorable for LA DDSs [48]. Osmotic pumping is another method of drug release, where osmotic pressure drives the influx of water into the non-swelling segment of the DDS, resulting in drug release [49]. 

Interestingly, the prevalent drug release pattern from LA DDSs is the triphasic rather than monophasic drug release pattern [41]. The first phase of drug release from an LA DDS demonstrates an initial burst release due to the rapid release of drug molecules located near the surface of the DDS or the surrounding tissue [50]. The extent of this burst release is influenced by factors such as the design and morphology of the DDS, polymer properties, fabrication process, storage conditions, and homogeneity of the drug–matrix concentration [50]. While an initial burst release may be desired for rapid drug action, it can significantly reduce the drug depot concentration, consequently impacting the DDS’s longevity. Strategies such as hydrophobic polymer coating on the outer surface of the DDS can curtail this challenge [50]. The second phase of drug release involves a slow-release period, where the drug diffuses through the polymer matrix. This phase concurrently occurs with polymer degradation in biodegradable systems [41]. The third phase may exhibit faster release as bulk erosion of the matrix starts. Drug release can also follow a biphasic trend, transitioning from the initial burst release to the zero-order kinetics [51]. Overall, the kinetics of drug release are complex and influenced by multiple factors. Understanding and optimizing the release profile of DDSs are crucial for achieving desired therapeutic outcomes and ensuring the efficacy of DDSs.

Molecular weight (MW) and the molar ratio of the monomeric units of a polymer play a crucial role in determining its physicochemical properties, including solubility, crystallinity, glass transition temperature (T_g_), and mechanical strength. Polymers with less elasticity result in non-deformable matrices and smaller pore formation, leading to a slower drug release [52]. Moreover, the degradation of the copolymer is influenced by the nature and molar ratio of monomeric units [53]. For example, a higher molar ratio of hydrophilic monomer, glycolic acid (GA), in PLGA composition leads to its faster biodegradation and subsequent drug release [54]. 

Polymer crystallinity refers to the proportion of crystalline and amorphous regions within the polymer structure [55]. The ratio of different monomeric units in a copolymer affects the polymer’s crystallinity and T_g_. Most polymers are semicrystalline in nature, in which the amorphous domains separate the crystalline domains. The water permeability of the polymer is primarily controlled by the percentage of crystallinity. An increased level of polymer crystallinity decreases the overall water permeability, resulting in a slower rate of polymer biodegradation and a decrease in the drug release rate [56]. In high-MW polymers, the influence of crystallinity on drug release depends on the presence of monomer crystallinity [57]. A notable example is the biodegradability of PLGA, where the ratio of its monomers, lactic acid and GA, determines the degree of crystallinity, subsequent biodegradation, and drug release profile. PLLA, composed of L-lactic acid, is highly crystalline, while PDLA, composed of D-lactic acid, is completely amorphous. Like PLLA, PGA is also a highly crystalline polymer. The degree of crystallinity and biodegradability of PLGA copolymer depends on the molar composition of lactic acid and GA comonomers in the copolymer [58]. Furthermore, the ratio of the amorphous and crystalline regions also influences the polymer’s T_g_, which in turn affects its mechanical strength and water permeability. For example, a polymer with T_g_ equal or close to physiological temperature can transform into a rubbery state, facilitating water diffusion and promoting drug release [44]. 

## 4. Common Polymers in LA DDSs

Advancements in synthetic polymer chemistry have led to the development of numerous biocompatible and biodegradable polymers. Synthetic polymers are derivatized from chemically synthesized monomers and are typically biologically inert, causing minimal adverse immune reactions. They also exhibit more predictable physical and chemical properties compared to natural polymers [59]. The ability to adjust the physicochemical properties of synthetic polymers allows for tailored drug release kinetics, making them more favorable than natural polymers [60]. Polymers used in the fabrication of LA DDSs can be categorized as either biodegradable or non-biodegradable [61]. Injection site reactions and requirement for removal of DDSs after the lifespan spurred the development of biodegradable polymers. The use of biocompatible and biodegradable synthetic polymers in LA DDSs eliminates the need for removal after their lifespan. In the following discussion, we will explore the polymers commonly employed in LA DDSs, along with their physiochemical characteristics and mechanisms of drug release.

### 4.1. Polyethylene Glycol (PEG)

PEG, also known as macrogol, is synthesized by ring-opening polymerization of ethylene oxide. Its outstanding biocompatibility, hydrophilicity, non-immunogenicity, and antifouling properties towards serum protein contribute to its extensive use in DDSs [62]. Chemical modification with PEG, commonly referred to as PEGylation, enables the conjugation of biomolecules to the surface of micro- or nanoparticles. Surface PEGylation of DDSs substantially reduced its opsonization by decreasing the chances of protein corona formation, which eventually increases the plasma apparent half-life and reduces the immunogenicity in vivo [63,64]. Based on structure, PEG can be classified as linear, branched, and star-shaped [65]. The end hydroxyl group of the PEG can be transformed into various active functional groups, such as amino, maleic amide, and carboxyl moieties, suitable for functionalization with various drugs and other pharmacologically active biomolecules [66,67]. However, the minimal availability of active functional groups in linear PEG offers limited PEG–drug covalent conjugates, resulting in low drug-loading content. Therefore, employing a four- or eight-arm PEG structure can increase the number of active end functional groups, resulting in improved drug loading in DDSs [68,69]. PEG cannot undergo biodegradation; hence, a low-MW PEG has been recommended for use in DDSs so that it can easily be eliminated from the biological system. In addition, PEG can be copolymerized with biodegradable polyesters, such as PLA or PLGA, to improve the overall biodegradability of the copolymer [70]. 

### 4.2. Poloxamers

**Figure 2:**
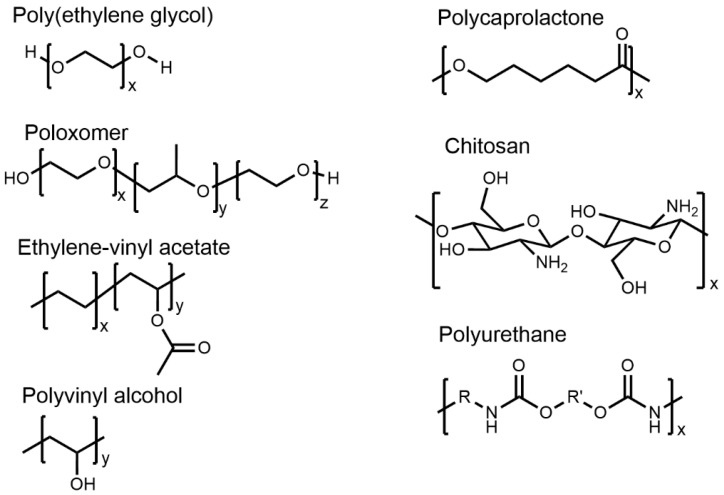
Chemical structures of the polymers used in LA delivery systems.

Poloxamers are proprietary polymers of BASF, also known as Pluronics^®^. These are triblock copolymers and have been synthesized by sequential polymerization of ethylene oxide (EO) and propylene oxide (PO) (Figure 2). The arrangement of blocks in the copolymer can either be PEO-b-PPO-b-PEO or PPO-b-PEO-b-PPO [71]. Poloxamers are amphiphilic in nature because of the hydrophilic and hydrophobic properties of PEO and PPO, respectively. Amphiphilic poloxamers self-assemble into a spherical micelle at a concentration above their critical micelle concentration (CMC) at a specified temperature. These micelles adopt a core–shell morphology with a PPO block in the core and PEO block on the shell. Some poloxamers can transform their morphology from micellar structures to solid micellar cubes, producing thermo-responsive clear and rigid hydrogel at elevated temperatures and concentrations [72]. For example, Poloxamer 407 can form hydrogels in 20% *w*/*w* solution [73]. The rate of drug release from the poloxamer-based DDSs primarily depends on the drug’s hydrophilicity and relative block lengths of PPO and PEO [74,75]. It has been seen that hydrophilic drugs typically followed diffusion-induced release, whereas hydrophobic drugs followed erosion-induced release [76]. In addition, factors such as poloxamer concentration, lipophilicity, physicochemical properties of the used solute, and the amount of the aqueous channel network in poloxamer gel significantly influence drug release. In an abundant aqueous environment, the structure of poloxamer micelles breaks down quickly, leading to gel matrix degradation [77].

### 4.3. Ethylene-Vinyl Acetate (EVA)

The random copolymer of ethylene (E) and vinyl acetate (VA) is often referred to as ethylene-vinyl acetate (EVA) (Figure 2). The copolymer has been synthesized by radical copolymerization of ethylene and vinyl acetate with various feed molar ratios of monomers depending on its applicability [78]. EVA has been extensively used in clinical settings, especially for ocular implants, contraceptives, and hormone replacement therapy. NuvaRing^®^, Progestasert^®^, and Implanon^®^ are the three most widely used EVA-based contraceptive products, where EVA acts as a release-rate-controlling membrane [79]. EVA has also been used in the delivery of buprenorphine/hydromorphone for treatment of opioid withdrawal.

Like other polymers, EVA’s release kinetics is influenced by its physicochemical properties, such as hydrophobicity, crystallinity, etc. Furthermore, EVA’s physicochemical properties have been governed by wt% of VA. The commercially available EVA contains 1 to 40 wt% VA, randomly distributed throughout the EVA backbone [80]. The acetate side chain of VA creates steric hindrance that restricts the alignment of the polymeric chain. As a result, an increasing percentage of VA decreases the crystallinity [81]. Eventually, EVA becomes nearly amorphous when the VA content increases to 40 wt% [82]. EVA exhibits a complex combination of amorphous and crystalline phase regions, characterized by at least two T_g_ (between −35 to −25 °C), which is barely influenced by the overall VA content [83,84]. In addition, the MW distribution, branching, and morphology also have a prominent impact on the physicochemical properties of EVA. In hydrogel reservoir-based delivery systems, EVA is commonly employed as a membrane for controlling the release rate. Additionally, EVA finds extensive application as an excipient in the development of nano- and microparticles as it is compatible with a wide range of excipients that can be used in complex multilayer polymer blends to further tune the release rate [79,85].

### 4.4. Polyvinyl Alcohol (PVOH)

Polyvinyl alcohol (PVOH) is a highly biocompatible and biodegradable semicrystalline polymer used in LA DDSs [86]. PVOH is synthesized by sodium-hydroxide-mediated hydrolysis of polyvinyl acetate and can be classified as fully or partially hydrolyzed PVOH [87,88]. PVOH is highly water soluble because of its pendant hydroxyl groups (Figure 2). The solubility, mechanical, and adhesion properties of PVOH depend on its MW and percentage of hydrolysis. Partially hydrolyzed PVOH (87–89%) shows improved solubility, flexibility, and adhesion to hydrophobic surfaces, while highly hydrolyzed PVOH (91–99%) exhibits enhanced water stability and increased tensile strength and adhesion to hydrophilic surfaces [89]. The hydroxyl groups in PVOH can form inter- and intramolecular hydrogen bonding, which influences its rheological, viscoelastic, and solution properties. PVOH’s characteristics, including high surface stabilization and chelation, make it an excellent candidate for drug delivery [90,91]. PVOH has been used in many clinically approved products over the last few decades. For example, the Iluvien^®^ implant has been fabricated by using PVOH and silicone and used for macular edema [92]. In addition, PVOH has been found to be extensively applied in preclinical research for developing hydrogels, microspheres, and nanoparticles for various therapeutic purposes [86]. PVOH hydrogels can be physically or chemically cross-linked, holding drug molecules in their 3D cross-linked structure [93,94]. These hydrogels have gained popularity as the drug release kinetics from these hydrogels can be predicted by observing water uptake and swelling index [93,95].

PVOH has demonstrated remarkable biocompatibility in various studies conducted since the late 1980s, as evidenced by its successful use in biomedical applications [96,97]. Examples include its inclusion in ophthalmic solutions and tear replacement solutions without causing any discomfort to the eyes [98]. In recent years, PVOH hydrogels have found extensive use in cartilage replacement, tissue sealants for emphysema treatment, wound dressings with healing agents, and as a barrier against secondary infections [99,100,101]. The toxicity profile of PVOH was extensively evaluated after oral and intravenous injections of PVOH in animal models [102,103]. Oral administration of PVOH to rats demonstrated no reproductive, neurological, or systemic toxicity [104]. Acute oral toxicity of PVOH was found to be very low with poor gastrointestinal absorption and no mutagenic or clastogenic effects [102]. The highest oral dose with no observed adverse effect level (NOAEL) was determined to be 5000 mg/kg/day [102]. After oral administration, PVOH was primarily excreted in feces and minimally in urine [102]. The liver was identified as a principal organ for PVOH deposition, resulting in its slow elimination from the body [105]. Furthermore, genotoxicity tests indicated no evidence of mutagenicity or carcinogenic activity [106].

### 4.5. Polyurethanes (PUs)

PUs are condensation polymers, synthesized by step-growth polymerization of isocyanates and polyols. Chain extenders are often added during the synthesis process of PUs to enhance their mechanical strength. The stiffer hard segments in PUs are made up of chain extenders and isocyanates, and flexible soft segments of PUs are made up of polyols. The interplay between these segments endows polyurethanes with a balance between rigidity and elasticity [107]. Both aliphatic and aromatic diisocyanates are employed for the synthesis of PU. PUs derived from aliphatic diisocyanates tend to be more biocompatible and show enhanced resistance to biodegradation than those produced from aromatic diisocyanates [108,109]. Polyether and polycarbonate are commonly employed as polyols, with MW typically ranging from 1 to 5 kDa. Notable advancements were made in developing biodegradable polyester-based mechanically strong PUs, widely used in manufacturing VR-like implants [110]. For instance, a biodegradable intravaginal ring was developed using a poly(ester urethane) that released antiretroviral DPV for more than a month [111]. PUs derived from polyethers display enhanced hydrolytic stability and remain elastic at low temperatures. However, they are susceptible to both oxidation and thermal degradation. The addition of antioxidants has further increased their stability. For example, PEG, a widely used hydrophilic polyol, significantly influences the drug release rate from PU-based DDSs [112,113]. 

The drug release from the PU-based DDSs has three primary mechanisms. These include solute diffusion, polymer swelling, and polymer degradation [114]. PU-based DDSs show an initial burst release which is mostly because of solute diffusion through PUs. In non-degradable PU-based DDSs, the drug release rate is determined by the thickness, swelling capacity, and permeability as well as the drug concentration gradient and polymer–drug compatibility [43]. In biodegradable PU-based DDSs, the release rate is determined by its own swelling capacity and rate of degradation or erosion. The PU can be hydrophilic or hydrophobic depending on the nature of the diol/polyol and chain extender employed during synthesis. Unlike hydrophobic PUs, hydrophilic PUs can easily swell and demonstrate a faster rate of drug release. For example, the rate of DPV release from swellable PU-based DDSs is significantly higher than its non-swellable counterpart [115]. Hydrophobic PUs tend to experience surface erosion, whereas hydrophilic PUs with fewer reactive ester linkages typically undergo bulk erosion [116,117]. The type of diol/polyol used in PU-based DDSs determines its swelling capacity which in turn controls its rate of degradation/erosion. The degradation of PUs entirely depends on the hydrophilicity and biodegradability of diol/polyol together with the chain extender [118,119]. PUs with PEG as diol can easily be hydrated and the rate of degradation is accelerated. Moreover, PUs can also undergo chemical degradation or enzymatic degradation [120]. 

### 4.6. Polyesters

Biodegradability of polyesters, such as PGA, PLA, and PLGA, makes them the most extensively used polymers in LA DDSs. These polymers serve as vehicles for delivering various pharmaceutically active biomolecules like proteins, nucleic acid, small drug molecules, etc. PLA is synthesized through lactide ring-opening polymerization. The physiochemical properties and release profile of PLA can be tuned by tailoring the degree of racemization of lactic acid. PLLA, derived from L-lactide, exhibits a semicrystalline structure characterized by its hardness and transparency, with a T_g_ of 53 °C. In contrast, PDLA, derived from D-lactide, is amorphous in nature, displaying lower mechanical strength and a T_g_ of 55 °C [121]. Due to the high thermal stability of PLA, it can be fabricated using various thermal processing methods, such as injection molding, extrusion, etc. PLA degrades through the hydrolysis of its ester bond and forms lactic acid as an intermediate degradation product which is further converted to carbon dioxide and water, a biocompatible and non-toxic end product [122]. Moreover, the intermediate lactic acid can accelerate the degradation by catalyzing the degradation process. Biodegradation of PLA is very slow due to its semicrystalline nature. Furthermore, side chain methyl group cause hydrophobicity in PLA which further lowers its rate of degradation. Therefore, depending on the implant size and MW used, PLA can take as long as 2 years to degrade inside the body [121]. The slow biodegradation rate of PLA has been utilized in the development of Brimo DDS^®^, an intravitreal implant that incorporates PLA for the sustained release of brimonidine to treat geographic atrophy [123]. 

PGA is another polyester. PGA is synthesized by the ring-opening polymerization of glycolide [46]. In comparison with PLA, PGA is highly crystalline, providing it with notable mechanical properties that include enhanced strength, rigidity, and thermal resilience [124]. However, PGA has limited potential as a standalone polymer for several reasons. First, the hydrolysis of the ester in PGA is faster than PLA due to its high hydrophilicity. Additionally, the degraded byproduct, glycolic acid, can potentially cause inflammation [125]. Second, PGA possesses inherent brittleness and limited solubility in commonly used organic solvents which further restricts its processibility to formulate a desired DDS. Therefore, PGA is frequently employed in combination with other polymers to overcome any usage limitations. 

PLGA, one of the extensively utilized polymers in LA formulations, comprises a substantial 46% of the approved LA injectable medications made from polymers [126]. PLGA is synthesized by a combination of ring-opening and condensation polymerization reactions. PLGA can be synthesized in various MWs and lactide-to-glycolide (L/G) ratios, where an increase in the L/G ratio corresponds to a slower rate of polymer degradation. For instance, in aqueous conditions, PLGA polymers with L/G ratios of 50:50, 75:25, and 85:15 have degradation times of approximately 1–2, 4–5, and 5–6 months, respectively [121]. 

Drug release from PLGA or PLA-like polyester mainly has two mechanisms: diffusion and bulk erosion or degradation [81]. At the early stage of release, diffusion is the main release mechanism which depends on the concentration gradient and the shape of the DDS [41]. Over the time, PLGA erosion becomes the dominating mechanism. The drug release process from PLGA-based DDSs starts with slow water infiltration followed by swelling of polymer. The swelling of the polymer core increases the pore’s size and makes way for the drug molecule to diffuse through the polymer matrix or membrane [127]. Soon after, PLGA undergoes degradation, eventually forming lactic acid and GA as byproducts. These acids act as auto-catalysts and further speed up the degradation process [41]. Collectively, the MW, PLA:PGA ratio, and geometry of the PLGA-based DDS influence the water absorption and, eventually, its degradation. Moreover, PLGA–drug interaction, drug solubility, pH of the surrounding environment, osmolarity, and porosity of the DDS have a considerable impact on the release rate. PLGA-based DDSs mostly have triphasic release kinetics [41].

Polycaprolactone (PCL) is a semicrystalline and hydrophobic polyester which is typically synthesized by the ring-opening polymerization of ε-caprolactone. Compared to other hydrophilic polyesters, PCL shows a very slow rate of biodegradation [128]. Microorganisms can slowly degrade PCL by using lipase, an ester hydrolysis enzyme. However, the hydrophobicity limits the interaction between PLA and lipase, therefore chemical degradation remains the primary degradation mechanism [129,130]. PCL-based DDSs can maintain a sustained drug release over several months. PCL is also known for its mechanical strength, which has made it a popular choice in applications like surgical dressing, wound healing, and contraceptive implants [131]. By taking advantage of the compatibility between PCL and various hydrophobic drugs, the drugs can be uniformly dispersed in the PCL matrix [132]. Additionally, it can be blended with an array of both natural and synthetic polymers, including starch, hydroxyapatite, chitosan, PEG, PU, oxazolines, PEO, and PVA. The blending allows fine-tuning of PCL’s mechanical strength, crystallinity, and degradation behavior and accomplishes an ideal DDS [133].

For PCL-based DDSs, hydrophobicity and crystallinity are two important factors that dictate the degradation and drug release kinetics. The chemical degradation of PCL-based DDSs happens in two steps. The first step is the rate-limiting step, where water infiltrates and hydrolyzes the amorphous region, which causes mass loss and is followed by an increase in porosity in the DDS. In the second step, a PCL degradation product, 6-hydroxyhexanoic acid, further catalyzes the hydrolysis process which eventually propagates to the crystalline region of the PCL [132]. Interestingly, the degraded form of PCL can be phagocytosed by cells [131]. PCL-based DDSs usually have biphasic release kinetics, where there is an initial burst release of the drugs followed by a sustained release phase. It has been evidently seen that the initial burst release is much higher for hydrophilic drugs than for hydrophobic drugs [109,134,135]. 

### 4.7. Chitosan and Hyaluronic Acid (HA)

Chitosan, a natural biopolymer, has widely been used in tissue engineering, bioscaffold creation, and drug delivery application. Chitosan has been derived from chitin (a long-chain natural polysaccharide) by adopting the alkaline or enzymatic deacetylation process [136,137]. Chitosan is a copolymer of D-glucosamine and N-acetyl-D-glucosamine linked through β-(1→4)-glycosidic bonds, giving it a rigid crystalline form. The chitosan backbone contains ‘–NH_2_’- and ‘–OH’-like active functional groups that can be easily functionalized with active biomolecules, making it popular for nano- or microparticle-based DDSs. Chitosan, a biodegradable polysaccharide, is particularly notable for its versatility in sustained drug delivery [138]. The physicochemical characteristics of chitosan depend on its MW and degree of acetylation. Chitosan exhibits low water solubility, enabling it to remain in the body for extended periods. This property has made it particularly useful for sustained release in oral drug delivery applications [139]. Moreover, inspired by anti-inflammatory, antibacterial, and mucoadhesive properties, chitosan has been widely used in wound dressing, healing, and pulmonary DDSs. 

HA is a naturally occurring non-immunogenic and hydrophilic polysaccharide. It is commonly used in the development of DDSs. The HA contains two basic sugar units, namely, D-glucuronic acid and N-acetyl-D-glucosamine, conjugated through β(1–4) and β(1–3) glycoside bonds [140]. HA is known to interact with CD44 cell surface receptors, and exogenous HA can reduce HIV-1 infection of CD4+ T-cells in a CD44-dependent manner [140]. However, endogenous HA failed to do so as it undergoes a rapid degradation at the cell surface by the enzyme called hyaluronidase. Moreover, HA is one of the major components of mucosa, which results in low mucoadhesive properties of exogenous HA. The thiol group (SH) modification on the backbone of HA can further increase its mucoadhesive property. In this context, Agrahari et al. formulated TFV-encapsulated nanofibers (NFs) by using thiolate hyaluronic acid to stop vaginal transmission of HIV [141]. The NFs were demonstrated to be safe toward the genital tract and other major organs of 57BL/6 mice. The NFs show hyaluronidase-responsive TFV release, resulting in the prevention of vaginal HIV transmission.

### 4.8. Polymers and Polymer Blends

Over the past few decades, research into LA DDSs has shown significant strides forward. The introduction of novel polymers and polymer blends has improved the efficiency of the existing drug delivery systems. This includes better biocompatibility, reductions in local and systemic toxicities, and optimal release profiles. The FDA-approved ISFI is one of them, which employed dimethyl sulfoxide (DMSO) and N-methyl-2-pyrrolidone (NMP)-like organic solvents to lower the solution viscosity of PLGA and induce phase conversion. Compared with DMSO, NMP has more carcinogenic, teratogenic, and mutagenic toxicities [89,142,143]. Therefore, to avoid NMP and minimize the use of PLGA, poly(3-hydroxybutyrate-co-3-hydroxyvalerate (PHBV) and poly (ethylene carbonate) (PEC) were developed. 

PHBV is a hydrophobic, thermoplastic, semicrystalline, biocompatible, and biodegradable linear aliphatic polyester that has been synthesized by incorporating 3-hydroxy valerate units into the poly(3-hydroxybutyrate) (PHB) [144]. PHBV is very brittle and has a melting temperature lower than PHB [145]. The thermal properties of PHBV can be improved through blending with PCL or TiO_2_ nanoparticles [146]. PHBV was used to formulate an aripiprazole (an antipsychotic drug)-encapsulating nanoporous ISFI [147]. Rheological analyses demonstrated the existence of a highly cross-linked network structure in the PHBV ISFIs. Spectroscopic analysis revealed the intactness of aripiprazole in the network structure. The ISFI demonstrated a burst release followed by a sustained release of aripiprazole over 18 days.

PEC is a biodegradable polymer synthesized by copolymerization of CO_2_ with ethylene oxide [148]. Generally, PEC is characterized by high ethylene carbonate content with low polydispersity and high glass transition temperature. PEC undergoes biodegradation through surface erosion, followed by the formation of ethylene glycol as a degradation product [149]. The in vivo biodegradability of PECs is influenced by the presence of reactive oxygen species, which are associated with inflammatory cells. Moreover, the molecular weight and percent of ether contained in PEC also have an immense effect on its degradation rate [150]. An LA ISFI was successfully demonstrated using PEC as a rate-controlling polymer and bovine serum albumin as a model drug. To compare the effects of DMSO and NMP on the microstructure and drug release of PEC-containing ISFIs, a bovine serum-albumin-encasing ISFI containing either PEC/DMSO or PEC/NMP was developed [151]. In comparison with the PEC/NMP ISFI, the PEC/DMSO ISFI demonstrated a quick release of DMSO followed by the formation of a depot with low porosity, which minimizes burst drug release. 

The emergence of stimuli-responsive polymeric biomaterials demonstrates improved spatiotemporal drug release. These smart biopolymers are responsive to various internal and external stimuli, including pH, redox potential, temperature, and enzyme activity [152,153,154]. Phenylboronic acid (PBA)-containing polymers are an example of such smart biomaterials which are sensitive to pH, sugars, and reactive oxygen species [155]. PBA-conjugated polymers have proven to be effective in pH-responsive delivery of anticancer and antiretroviral drugs. For instance, doxorubicin-encapsulating, PBA-modified poly(maleic anhydride)-F127 micelles show a pH-responsive release [156]. The use of PBA-conjugated polymers in LA ARV formulations could offer enhanced pharmacologic benefits. In this circumstance, a PBA-based mucin-like polymer system that blocks HIV-1 migration was developed within the genitourinary system [157,158].

## 5. LA ARV Delivery

**Figure 3:**
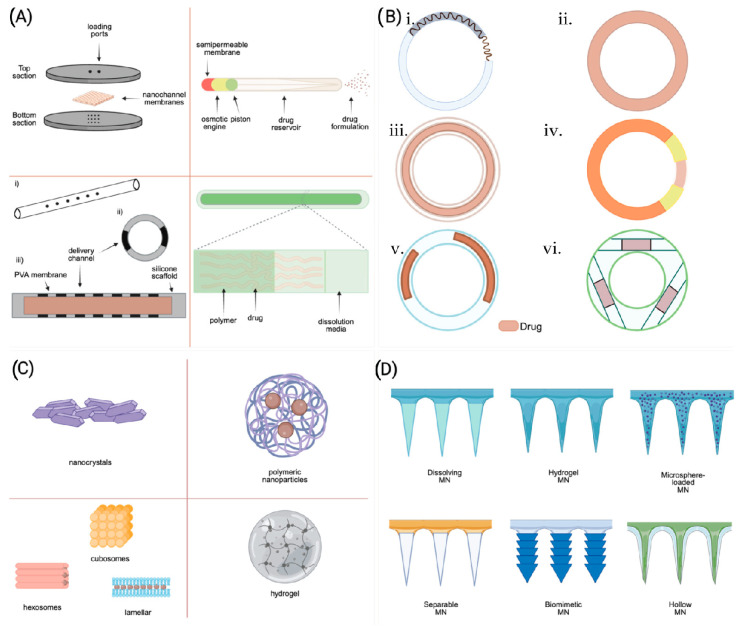
Different types of LA DDSs that are in either clinical or preclinical development. (**A**) Different types of solid implants. Reproduced with permission from Ref [165]. Copyright 2018 Antimicrobial Agents and Chemotherapy. (**B**) Different types of Vaginal rings—(**i**) over-molded metal spring design, (**ii**) matrix-type VR, (**iii**) Full-length reservoir-type VR, (**iv**) sandwich-shaped VR, (**v**) Partial reservoir-type VR, (**vi**) Insertable reservoir-type VR. Images were adapted under the terms of Creative Commons Attribution—Non-commercial from ref. [166]. (**C**) Nanocrystal formulations, polymeric nanoformulations, liposomes, and hydrogels. (**D**) Different types of microarray patches. Reproduced with permission [21].

LA DDSs, such as implants, VRs, MNs, and depot-forming nanoformulations, offer a promising avenue for extended release of ARVs beyond two months (Figure 3). In HIV-1-positive patients, the viral load is always persistent in tissue along with systemic circulation. This necessitates achieving continuous effective drug concentration both in the target tissue and plasma without reducing the longevity of the drug depot. Notably, for sexually transmitted diseases like HIV-1, precise drug concentrations within reproductive organs are pivotal for effective disease prevention. While local administration methods, such as VRs, offer a potential solution, they also face the challenges of maintaining the local therapeutic drug concentration without causing any toxicity to the surrounding tissues. Furthermore, the drug can be systemically absorbed, potentially diminishing local drug concentrations. In this context, implants or depot-forming injectables emerge as an alternative strategy as they can deliver the drug to reproductive organs through systemic drug exposure. Yet, achieving optimal drug concentrations without inducing local adverse effects in the implanted or injected site presents a multifaceted obstacle. The intricacies of polymer–drug interactions and device design play a pivotal role in dictating drug exposure and its release kinetics. Here, we will discuss recent advancements in LA ARV formulations, specifically focusing on the interplay between drug and polymer interaction and factors that influence the drug release kinetics. By deciphering the complex dynamics underlying polymer-mediated drug delivery, this exploration aims to pave the way to achieve an efficacious long-acting ARV formulation by fine-tuning their controllable parameters.

### 5.1. Implants

In 1938, Deansby and Parkes pioneered implantable drug systems by observing the effects of estrone pellet implants on male chickens [159]. Folkman and Long later refined this delivery system with a silicone rubber capsule for continuous drug release. This led to major developments in implantable drug delivery, especially for contraception and ocular treatments [159]. The approval of various contraceptive and ocular implants boosted their acceptance and uptake by patients. This progress influenced implants’ development for HIV-1 treatment and PrEP. In the early 2000s, research focused on implants for the sustained release of ARVs. TAF’s potency and suitable physiochemical properties, such as hydrophobicity, MW, and solubility in polymer matrices, make it an ideal candidate for delivery through implants. Other drugs like ISL, CAB, DTG, and entecavir are also being explored for implant-based delivery [15,160,161].

The formulations and drug release mechanisms of implantable devices are multifaceted and varied, making their categorization challenging. Nevertheless, those designed for HIV treatment and prevention can be broadly divided into two types. The first includes preformed solid implants, which are made of either biodegradable or non-biodegradable polymers and require surgical or invasive procedures for subdermal placement [15,162]. The second type involves injectable in-situ-forming implants (ISFIs). ISFIs can be injected in the form of suspension or solution in subdermal spaces where solvent-induced phase inversion produces a drug-loaded implant [10,163]. Even though most ARVs and the polymers used in the fabrication of implants are FDA approved with established systemic safety profiles, local toxicity concerns that are either drug specific or associated with the nature of implant materials remain. Improved understanding of how ARVs and their metabolites interact with and are eliminated from the surrounding tissues and extracellular environments is of significance. For instance, high concentrations of TFV and TAF have shown significant implant site toxicities in some preclinical studies and significantly affected rapid development and translation of these devices [164]. Nonetheless, advancements are being made in implant design to regulate TAF release and stability within the device.

**Figure 4:**
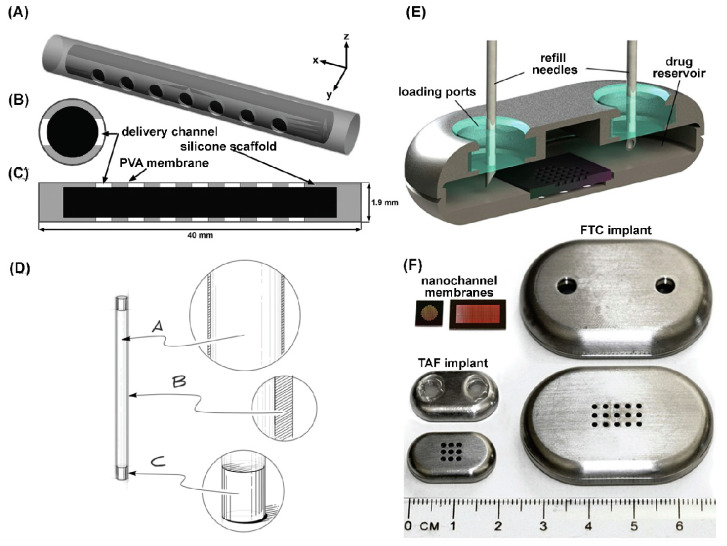
Different types of non-erodible implants are illustrated. (**A**–**C**) TAF implants contain silicone and PVA. Reproduced with permission from [15]. (**D**) PCL reservoir-style implant for TAF delivery, which comprises a formulated drug core (Reproduced under the Creative Commons CC-BY license from [12]). (**E**,**F**) Cross-sectional depiction of nanochannel-containing implant showing drug refill needles through the loading ports with resealable silicon plugs. TAF and FTC implants of two different sizes and shapes. Reprinted with permission [15].

A wide range of non-biodegradable (such as silicone and PU) and biodegradable polymers (PCL or PLA) have been used for ARV implant generation (Figure 4). Biomedical-grade silicones have been used for implant development for various diseases over six decades and are also the preferred materials for the development of ARVs, such as TAF implants. Irrespective of the extensive use of silicone-based TAF implants, TAF permeability through the silicone implant is low. To improve TAF permeability, implants have been fabricated with nano- or microchannels that aid in improved TAF release kinetics. To further control drug release rates, the implants were either coated with a rate-controlling membrane or the diameter and number of drug release channels were adjusted [15]. Interestingly, TAF release rate from this implant was independent of initial TAF loading. PK evaluation of this device in beagle dogs demonstrated a zero-order release kinetics of TAF (1.07 ± 0.02 mg/day) that sustained therapeutic TFV-DP levels in PBMCs for 40 days. The implant is currently in an early phase I clinical trial to assess its safety and tolerability [167]. The ability to remove the implant can be advantageous in the case of adverse reactions. However, removal of this device requires invasive surgical procedures that may limit broader use. In such cases, transcutaneously refillable silicone-coated nanochannels may offers enhanced pateint convinence and acceptability over conventional solid implants by offering refillability with explantation [11]. This design primarily comprises a transcutaneously refillable medical-grade titanium drug reservoir and a uniquely crafted silicone membrane housing TaN-coated slit nanochannels [11]. These nanochannels are arranged in square arrays and connected to the inlet and outlet of the membrane through a network of microchannels. Drug release rate from the implant is dependent on interactions with the interior surface of the membrane and can be further controlled by adjusting the number of nanochannels. 

Hydrophobic poly(tetramethylene oxide) subunit-containing PUs have also been used to create TAF implants [162]. As hydrophobic PU demonstrates minimal swelling, the drug inside the hydrophobic PU tube forms a suspension encircled by an outer layer of saturated drug. The difference in concentration of these two layers drives the drug release rate, which follows zero-order kinetics. This release rate can be fine-tuned by modifying the membrane wall thickness or composition of PU. PU-based pellets-in-a-tube-type TAF implants where a mixture of TAF, NaCl, and magnesium stearate were converted into a pellet and impulse sealed into PU tubes were created [162]. In this report, the release kinetics of TAF from the implant were not zero order. Interestingly, incorporating a hydrophilic excipient like PEG into the TAF pellet or increasing the crystallinity of the PU membrane had been found to enhance the drug’s release from the implant [13]. However, preclinical in vivo, safety, and tolerability studies in different animal models demonstrated that the PU-based TAF implant results in local inflammation and toxicities. The local toxicities observed in those implants may be the result of high TFV and TAF concentrations in the implant site, as observed in those studies. A similar implant design has also been employed to develop CAB implants [168]. In a comparative study, the release rate of CAB from various elastomeric PU membranes, including poly(dimethylsiloxane), poly(ethylene-co-vinyl acetate), and hydrophilic poly(ether) urethanes, was tested. Interestingly, only the segmented hydrophilic poly(ether) urethanes demonstrated a sufficient CAB release rate to reach a pharmacologically relevant concentration. Furthermore, using polyvinylpyrrolidone (PVP) instead of PEG as an excipient caused less mass swelling and higher implant drug release [168].

Biodegradable polymers are widely used for the development of reservoir-type TAF implants. PCL are TAF compatible. When a TAF-containing PCL implant is inserted subcutaneously, the biological fluid from the surroundings slowly penetrates the PCL implant, creating water-filled channels and dissolving TAF to facilitate its release. As more water enters the implant, it induces the bulk erosion of PCL. However, PCL’s bulk erosion rate is so slow that it can persist for up to a year [169]. A biodegradable PCL-containing solid TAF implant was recently developed [12]. This implant was 2.5 mm in diameter but of varying thicknesses and produced by a hot-melt extrusion process. The PCL tube was filled with a formulation containing TAF and castor oil and sealed at both ends through heat or injection sealing. The TAF release rate from the PCL implant depended on factors like the implant’s surface area, PCL’s MW, membrane thickness, and TAF solubility. TAF release was found to correlate with the implant’s surface area and inversely with PCL wall thickness [12]. Interestingly, thinner-PCL-wall implants demonstrated a rapid initial TAF release, while high-MW PCL implants released TAF more quickly. The effect of PCL’s MW on release rates was common in thinner walls rather than thicker ones. This is possibly due to differences in crystallinity between PCL types [12]. Incorporation of hydrophilic excipients in a PCL implant can increase the TAF release rate. For example, a PCL implant, where TAF was formulated with PEG_3350_ at various weight ratios, increased TAF dissolution from the implant. This leadsto rates increases of TAF [160]. The PEG_3350_. This eventually increases the drug release rate. It has been reported that the rate of TAF release is proportional to the mass of the PEG_3350_ used and the surface area of the implant.

ISL, a novel nucleoside reverse-transcriptase translocation inhibitor (NRTTI) has gained attention in implant generation due to the potency and extended intracellular apparent half-life of its triphosphate metabolite [14,170]. ISL was incorporated in both biodegradable and non-biodegradable polymer-containing implants for extended release [165]. Two types monolithic matrix-type implant were developed by using either biodegradable PLA and PCL or non-degradable EVA polymer for SC delivery of ISL [165]. The uniform dispersion of crystalline ISL in the polymer matrix resulted in a minimal implant surface porosity, which limited the water infiltration to the implant. Therefore, the implant showed a small transient initial burst release followed by a slow first-order release of ISL. The initial small transient burst release was presumably because of the rapid dissolution of ISL persisting on the implant surface. Furthermore, the rate of ISL release showed an increasing trend with its loading content. Several clinical trials involving ISL implants and oral regimens have been halted as reductions of the participants’ CD4+ T-cell and lymphocyte counts have been reported [167]. 

Given the instability and safety concerns associated with TAF and ISF-based implants, alternative approaches such as the development of stable TFV prodrugs and salts, new ISF analogs, ISFIs, and other potent drugs from different classes such as CAB and DTG (INSTIs) have gained considerable attention. In-situ-forming implants (ISFIs) are injectable drug formulations that turn into solid/semisolid depots after administration [171]. A combination of the drug, excipient or release-rate-limiting agents, and water-miscible organic solvents have been used in the formation of ISFIs. N-methylpyrrolidone (NMP) and dimethyl sulfoxide (DMSO) are the commonly used water-miscible organic solvents used in ISFIs. After SC administration of ISFIs, the water-miscible solvent starts diffusing out from the implant, and surrounding water starts to infiltrate. Subsequently, there is an immediate phase separation of solvents, solidification of formulations, generation of microchannels for water influx, and eventually formation of the ISFI [172]. The drug release rate largely depends on the phase-inversion kinetics of the implant, MW and hydrophobicity of the used drug molecules, and biodegradability of the excipient [173,174,175]. 

Biopolymers such as hydroxypropyl methylcellulose, PLGA, PEG, poloxamers, PCL, and poly(N-isopropyl acrylamide) have been employed to develop ISFIs for various indications [176]. The selection of polymer depends significantly on the drug’s physicochemical properties and the desired release rate. Generally, a polymer’s solubility should be compatible with the drug’s solubility characteristics for optimal drug release kinetics [177,178,179]. PLGA was used as an excipient and NMP/DMSO as water-miscible solvent to formulate DTG and CAB ISFIs [10,180]. The authors investigated the impact of physiochemical properties of the polymer, used solvents, drug–polymer miscibility into the solvent, and polymer-to-solvent ratio on the drug release kinetics. 

**Figure 5:**
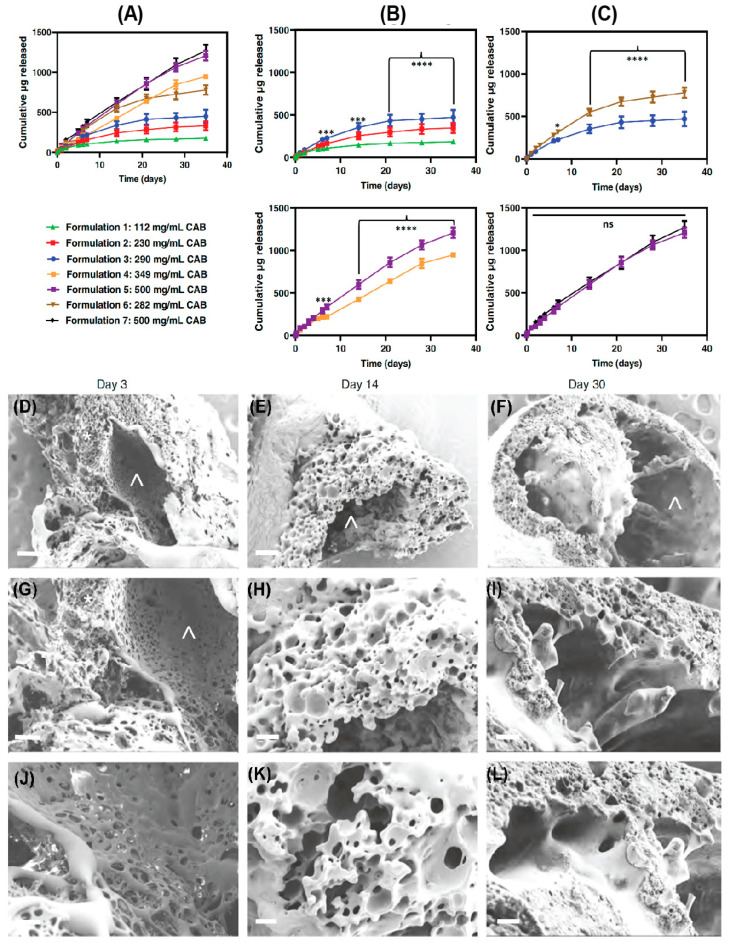
Microstructure in PLGA-based ISFIs: (**A**) Cumulative release of CAB ISFI PLGA formulations. (**B**) Effect of drug loading on CAB release. (**C**) PLGA molecular weight affects CAB release. To compare the drug release from different formulation at different timepoints in (**B**,**C**), two-way ANOVA with tukey’s multiple comparison test were performed. * *p* < 0.05, *** *p* < 0.001, **** *p* < 0.0001, and ns (not significant) when p > 0.05. (**D**–**L**) Effect of PLGA degradation on implant microstructure. SEM cross-section images of placebo ISFIs (1:2 *w*/*w* PLGA/NMP, PLGA MW 27 kDa) over a 30-day period. (**D**–**F**) Low-magnification image (100×) of the entire implant (scale bar = 100 µm). (**G**–**I**) Higher magnification (200×) of the implant shell (shell thickness was measured using SEM scale; scale bar = 50 µm). (**J**–**L**) Higher (500×) magnification of the center of the implant (scale bar = 20 µm). (**D**,**G**,**J**) Implants imaged at day 3 postincubation in 0.01 M PBS pH 7.4 at 37 °C. (**E**,**H**,**K**) Implants imaged at day 14 postincubation. (**F**,**I**,**L**) Implants imaged at day 30 postincubation. Symbol (*) represents implant shell. Symbols (^) represents the central pore of the implant. Images were reproduced under the terms of the Creative Commons CC-BY license [12,180].

The rate of drug release from the ISFIs is linked to the microstructure of ISFIs at the administration site. For example, scanning electron microscopy (SEM) imaging of DTG ISFIs demonstrated a central pore surrounded by a highly interconnected porous outer PLGA shell (Figure 5). Over time, the thickness of the outer PLGA shell decreased due to PLGA erotion, leading to an increase in the central pore size. The implant with a thinner shell and larger central pore size facilitated a high rate of drug release from ISFIs. The multispecies tolerability studies of ISFIs support their safety. The acceptability of ISFIs stems from their easy manufacturing and patient-comfortable administration process. Moreover, the clinical availability of leuprolide acetate ISFI (Atrix Laboratories Inc.) and that of Sanofi Aventis (Eligard®) supports the safety of this delivery system [181]. The overall limitations of ISFIs are injection site reaction, injection intervention, minimal control of the size and shape of the implant, and heterogeneous drug release [182,183].

### 5.2. Vaginal Rings (VRs)

Building on the VR’s clinical progress for contraception and hormone therapy, rings containing ARVs have been developed to prevent HIV-1 infection. In addition, VRs are gaining preference over other vaginal products. These include gels or creams based on their efficacy in time-dependent ARV delivery to the vaginal epithelium [33,184,185]. VRs are also widely accepted among women compared to other LA DDSs due to their user-friendly and discreet method of administration and easy reversibility of treatment when needed. The traditional approach to VR design involves uniform dispersion of drugs within polymer matrices. This is accomplished by molding a drug–polymer matrix into a ring-shaped device. Conventional VRs are classified into sandwich (or matrix)-type VRs and core (or reservoir)-type VRs [111]. In the sandwich design, a drug-dispersed polymer matrix is placed between two non-medicated rate-controlling polymer layers [166]. The core-type VR involves creating a small pellet containing a mixture of drugs and polymers. Several cores are combined into a single ring. The ring is covered by a drug-free secondary polymer membrane [166].

The drug release rate in VRs is typically governed by its drug diffusion through the polymer membrane [166]. When the VR is placed vaginally, water enters its wall, leading to polymer swelling and the dissolution of drugs [166]. The dissolved drugs near the matrix’s surface are diffused first. This action also forms a drug-depleted layer near the matrix surface. Subsequently, the remaining undissolved drugs undergo dissolution and diffuse through the polymer matrix. Factors such as the drug’s solubility in the polymer, the amount of drug loaded into the VR, and the surface area and thickness of the VR membrane influence this diffusion process. DPV, maraviroc, TAF, TDF, UC781, and MC1220 are being utilized in VR fabrication. Among these, DPV, TDF, and TAF, due to their ideal properties, have been predominantly studied for VR development. However, only the DPV VR has progressed to phase III clinical trials and received approval in several African nations for the prevention of HIV-1 [186].

Silicone, EVA, and PUs have been extensively used to develop DPV and TDF VRs [186,187]. They are clinically proven safe polymers for VR development. For instance, five of seven clinically approved VRs are made of silicone [111,188,189]. NuvaRing is the only VR that solely uses EVA, whereas another VR, Ornibel, uses PU as either a drug reservoir or a coating agent to control the drug release [186]. However, these polymers come with drawbacks. For instance, condensation-type silicone elastomers can produce a propanol byproduct. This can increase the drug solubility, prompting more solubilized drugs to move to the polymer surface. This then would lead to an increased initial drug burst release [190]. Furthermore, reports have emerged of biofilm formation due to enhanced bacterial adherence on EVA-based VRs [191,192,193]. However, biofilm generation can be significantly reduced by surface modifications such as in biocompatible surfactant, argon plasma treatment, or perfluoro-alkyl siloxane grafting on the elastomer [194,195,196,197]. 

The exploration of DPV VR development began nearly 25 years ago. The VRs were then developed using a condensation-cured silicone elastomer system [198,199]. To produce the core- or reservoir-type DPV VRs, DPV was blended with silicone elastomer and reaction injection molded at 50 °C to form an active core. This core was then segmented and enclosed within a non-medicated outer silicone membrane through another reaction-injection-molding process. The resultant VRs had a 5.50 mm and 9.00 mm outer cross-sectional diameter, while the total diameter of the VR was 55.00 mm. DPV release rate from the reservoir VRs depends on the solubility of DPV in the silicone matrix, and the diffusivity of the silicone solubilizes DPV through the non-medicated silicone sheath layer. In contrast, the matrix-type DPV VR was developed through a curing process involving DPV and silicone elastomer with a normal propyl orthosilicate cross-linker mixture. The implants did not show any significant local site reactions and proved to be well tolerated in the phase I clinical trial [198,199]. The PK and release kinetics of these two VR types showed notable differences. Matrix-type VRs exhibited a substantial initial burst release of DPV, reaching peak plasma concentration within 8 h. On the other hand, reservoir-type VRs took 10 days to reach the peak plasma concentration. Moreover, the average plasma concentration in matrix-type VRs was almost 50 times higher than in reservoir-type VRs. Interestingly, the matrix-type VRs demonstrated a rapid decline in plasma concentration over time, while the drop in plasma concentration from reservoir-type VRs was relatively slow [199,200]. From the clinical trial findings to date, the matrix-type silicone DPV VR stands out as the most effective one. There are several phase III clinical trials conducted with DPV matrix-type silicone-based VRs, and the DPV VR has been reported to reduce the risk of HIV-1 acquisition by 27% to 37% in African women [201,202].

Apart from DPV, TFV was the only other vaginal microbicide that demonstrated potential as a vaginal PrEP agent during clinical trials. As per the trial result, gel containing 1% TFV exhibited a 39% protection rate against HIV-1 infections in women [203]. Inspired by these findings, considerable research efforts were undertaken towards developing TFV or TFV prodrug-based VRs. While various polymers, including silicone, EVA, and PU, were investigated for creating TFV VRs, it was only the PU-based TFV or TFV prodrug VRs that demonstrated promising results in preclinical studies. For instance, Mesquita et al. developed silicone, EVA, and PU-based TDF VRs and compared the TDF release rate from these VRs [204]. The in vitro release study revealed that the release rate of TDF from a PU-based VR was 5 to 15 times higher in comparison to those created from EVA and silicone [204]. The differences in TDF release rate were attributed to the varying solubility of TDF in employed polymers. Specifically, TDF showed lower solubility in silicone and EVA as opposed to PU, resulting in a decreased diffusion of TDF through the polymer membrane [204].

The higher release rate of TDF from hydrophilic PU-based TDF VR has also been investigated [204]. Additionally, the authors compared both matrix and reservoir types of TDF VRs, made with hydrophilic polyether urethane (PEU). To prepare the matrix-type TDF VR, TDF-loaded hydrophilic PEU was extruded first using a twin-screw extruder and then cut into small pellets. Then, the pelletized segments were re-extruded, cut into 15.5 cm segments, and end-joined using induction welding [204]. For preparing a reservoir-type TDF loaded VR, the hydrophilic PEU was extruded into tubes, and the empty tubes were filled with either TDF only or a formulation of TDF and NaCl (86:14 wt% ratio) [205]. The matrix facilitated an immediate drug release, which then decreased by a factor of at least 20-fold from day 1 to day 28. In contrast, reservoir-type VRs showed a prolonged lag time to start the drug release. Interestingly, when an osmotic agent like NaCl was added to the reservoir-type VR, there was an increased influx of vaginal fluid into the VR, prompting a quicker TDF release. To establish human safety and tolerability, a phase I clinical trial was conducted using a reservoir-type HPEU TDF VR. However, the trial was discontinued due to the observed genital ulcers among the participants. However, no ulceration was observed among the participants who received placebo-containing VRs manufactured using the same technique, indicating the observed local adverse effect is not due to the polymer [17].

Hydrophilic PU (HPU) membrane-based reservoir TFV VRs were also reported [203]. The VR was produced by making an HPU tube by hot-melt extrusion and then filling the tube with either TFV or TFV/glycerol/water-containing semisolid formulation. The end of the tube was sealed with an induction welder. Only the TFV-containing VR showed a slow but steadily increasing TFV release rate in the in vitro release study. However, adding an HPU-permeable substance, such as glycerol with TFV in the polymer matrix, increased its solubility in HPU and release rate [203]. 

PU-based VRs were also used for the vaginal delivery of IQP-0528 and MIV-150. IQO-0528 is an NNRTI that has physiochemical properties similar to DPV. A matrix-type PU-based VR was developed to deliver IQP-0528 [206]. The VR was prepared by an injection-molding technique after mixing the drug and Tecoflex EG85A PU. In vitro studies with this VR indicated a linear relationship among the drug release rate, VR surface area, and drug loading to the VR. However, in vivo findings indicated that drug release was not solely determined by drug loading or VR surface area. Instead, it was primarily driven by diffusion, contingent on both the VR drug concentration and drug’s presence in the surrounding media [206].

### 5.3. Microneedles (MNs)

Microneedles (MNs), also known as microneedle arrays or patches, are transdermal drug delivery systems featuring tiny projections around 200 µm tall. While initially developed for immediate-release transdermal drug delivery, MNs have now evolved as innovative LA DDSs due to the advancement in polymer chemistry. MNs penetrate through the skin to the dermis, an area rich in blood vessels, ensuring efficient drug absorption while reducing systemic exposure and bypassing first-pass metabolism [207,208,209]. Their small size and length cause less pain, as pain receptors reside deeper in the skin. The design and composition of MNs, including tip length, spacing, diameter, and overall geometry, can be tuned to optimize drug delivery. Further, MNs can be combined with other drug delivery technologies like nano- or microparticles or hydrogels for extended drug delivery [210,211]. Their benefits have spurred a significant increase in research in recent decades. Particularly in ARV delivery, their discreet, easy, and minimally invasive nature is recognized and widely accepted [212]. For instance, a survey study was conducted to gather opinions from patients and healthcare professionals regarding the use of MNs for ARV delivery. The results showed a positive attitude towards MN technology, with both groups acknowledging the benefits of discreet and self-administrable MNs for administering ARV drugs [213]. Given the constraints related to permeability through the skin’s stratum corneum, MN technology proves particularly effective for potent, lipophilic, and low-MW drugs. ARVs such as RPV, CAB, and BIC have demonstrated significant potential when employed with MN technology. 

There are several types of MNs based on their design and drug-releasing mechanism. Each MN’s drug release kinetics varies based on the MN’s geometry, surface area, number of MNs per patch, polymer used, nature of the drugs, and its loading content [214]. In general, MNs are classified into solid MNs, drug-coated MNs, dissolving MNs, and hollow MNs. Solid MNs are usually used to pretreat the skin to create microchannels to facilitate drug absorption before applying any transdermal topical gel or formulation [214]. Polymers with good mechanical strength, such as silicone, PMMA, PCL, PLGA, PLA, and PGA, and metals such as stainless steel, titanium, and ceramics have been used to prepare solid MNs [215]. In drug-coated MNs, the outside of solid inert MN tips is coated with drug formulation. Upon insertion of the drug-coated MNs into the skin, the drug formulation is dissolved in the surrounding medium to release the drugs from MNs. These MNs are less utilized for LA DDSs because of their inability to form a drug depot. However, for LA drug delivery, dissolving MNs containing a nanocrystal formulation of ARVs have utility.

**Figure 6:**
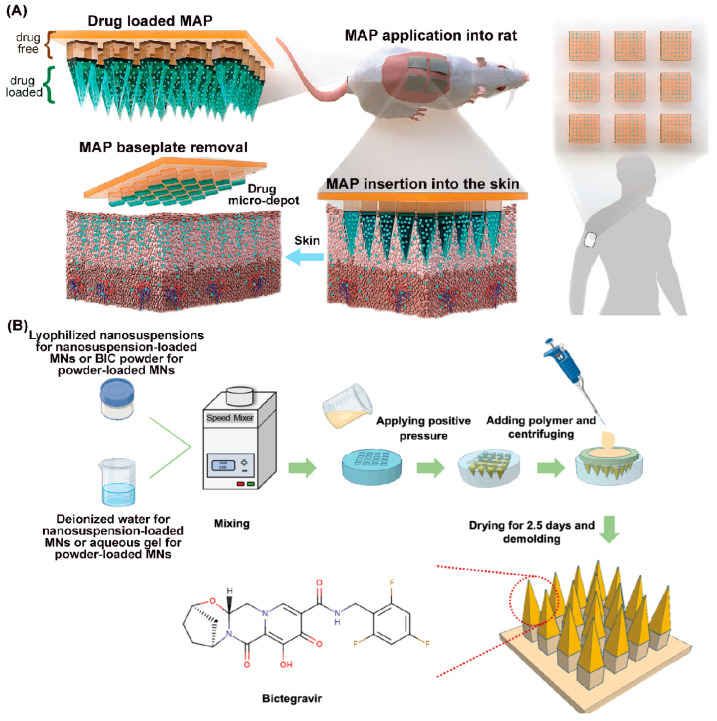
Fast-dissolving MNs have been made for ARV delivery. (**A**) Schematic depiction of programmed dissolving bilayer MAP with a high drug loading of LA microdepots for potential monthly human application. (**B**) Schematic presentation of the MN preparation process. The figure was reproduced under the terms of the Creative Commons CC-BY license [22].

Dissolving MNs are made of biocompatible polymers that can encapsulate drug-containing matrices and, upon administration, dissolve in the biological fluid and release the drug content [216]. Nanocrystal suspension of ARVs, such as CAB, RPV, and bictegravir (BIC), when delivered through dissolving MNs, can easily form dissolution-controlled drug depots (Figure 6). This strategy can allow a less painful way of self-administration of ARVs. The tips of dissolving MNs can be made of biodegradable or non-biodegradable polymer or sugar, and the rate of polymer or sugar degradation determines the MNs’ release duration [216,217,218,219,220,221]. The fast-dissolving MNs use hydrophilic polymers like dextrose, maltose, and chitosan, while slow-dissolving ones employ hydrophobic polymers like PLGA, PVA, and PLA, due to their biocompatibility, mechanical strength, and slow erosion [222,223,224]. PLGA MNs are mainly loaded with hydrophobic drugs and can be designed to separate from their baseplates upon insertion [223,225,226]. PCL is also used in MN production which allows controlled drug release using near-infrared light as an external trigger [227].

To create a bilayer dissolving MNs for transdermal delivery of RPV, a first layer or MNs tips were developed using varying compositions of PVP and RPV nanocrystal suspension [23]. The drug-free baseplate of the MNs was made using an aqueous solution of PVA and glycerol. The fast-dissolving MNs dissolved within a few minutes after the insertion into the skin and formed an RPV depot in the application site. However, the dissolution testing of MNs in excised skin demonstrated a slower dissolution profile of RPV suspension, which may be due to the hydrophobicity of RPV in the MN tips. Also, the concentration of RPV in the skin tissue was proportional to the MN application time. Although varying compositions of RPV nanocrystal suspension and PVP were used to manufacture MNs, the MNs with 70% RPV, 15% PVP, and 15% water demonstrated better plasma RPV levels compared to other MNs. Due to the discreet nature of the MNs and their ability for localized delivery of drugs, the same type of RPV MN was also generated by the same group for vaginal and CNS delivery of RPV [228,229].

A similar type of bilayer MN has also been developed by the same group for the transdermal delivery of BIC and CAB nanocrystal formulation (Figure 6). For CAB MN generation, bilayer MNs were developed, where the MN tips were generated by using a hydrogel composed of a 20:20:60 PVA:PVP:CAB LA nanoformulation [22]. The baseplate was made using PVA and PVP. The two polymer blends formed a sufficiently strong MAP tip, which could be attributed to the hydrogen bond formed between the carbonyl group of PVP and the hydroxyl group of PVA. This hydrogen bonding also improved the crystallinity and cohesiveness of the polymer. An in vitro MN tip dissolution study showed that CAB MN tips dissolved almost 100% within the first 60 min after administration. Importantly, after a single MN administration in rats, these MNs maintained CAB therapeutic levels for up to a month [22]. For developing BIC-containing MNs, first, a PVP- and PVOH-containing BIC nanosuspension was prepared and lyophilized. Then, the lyophilized powder was reconstituted in a very small amount of deionized water, and MN tips were generated from the resultant suspension. The baseplate of the MNs was made using PVP and glycerol. Similar to CAB MNs, BIC MNs also maintained plasma BIC levels equal to the human therapeutic level for up to a month [230].

Newer types of MNs, such nano- or microparticle-loaded MNs, hydrogel-forming MNs (HFMNs), and separable MNs, are also becoming very popular and useful in drug delivery [223,231]. HFMNs are particularly important for LA drug delivery and are made from swellable cross-linking polymers. In a dry state, HFMNs are sturdy enough to penetrate the skin. Upon insertion, HFMNs form hydrogels by soaking up water from the interstitial fluid, causing the formation of water-filled channels in the MN tips [232,233]. The drug’s release rate from HFMNs is influenced by the dimensions and number of channels, as well as the size of the drug particles [234]. Unlike dissolving MNs, HFMNs have a higher drug-loading capacity [235]. However, this capacity varies based on the polymer type and cross-linker used. Common polymers for HFMNs include PMVE/MA, PVA, EVA, chitosan, and silk fibroin, often cross-linked with hydrophilic polymers or compounds like PEG, dextran, and CMC [231,235]. The drug release rate can be tailored by adjusting the percent of cross-linker and swelling ratio.

### 5.4. Polymeric Micro- and Nanoparticles

Polymeric microparticles are microscopic particles with sizes of 1 to 1000 μm, where a core substance is encapsulated by one or more layers of the polymer membrane. Microparticles can further be divided into microspheres and microcapsules. While microspheres consist of a uniform matrix with the API dispersed throughout, microcapsules feature a distinct core, which can be liquid, solid, or semisolid, enveloped by a continuous polymer coating [236]. Nanoparticles have a similar feature but a size of 1 to 1000 nm. Micro- and nanoparticles can be prepared through a process called micro- or nanoencapsulation, respectively. This process entails encapsulating solid or liquid drug particles either dispersed or dissolved within a polymeric matrix or as a core surrounded by a polymeric shell. The polymer used in these particulate systems is alginate, chitosan, PLA, or PLGA. However, PLGA is the most widely used polymer for micro- or nanoencapsulation, which is used in around 50% of clinically approved LA injectables [237]. The proven clinical safety and tunable drug release kinetics make PLGA a widely used polymer in particulate drug delivery systems. The encapsulation of drugs in micro- or nanoparticulate systems can be achieved through either physicochemical or mechanical processes. 

The physiochemical process of drug entrapment involves coacervation or phase separation and inotropic gelation techniques [238,239]. There are three steps of microencapsulation by the coacervation-phase separation technique, which is carried out under continuous agitation. The first step involves the dispersion of the drugs in the polymer solution or suspension, then the coacervation process starts with a stimulus such as an addition of acid or salt (pH change), an input of non-solvent into the dispersion, or a temperature change in the emulsion which causes the precipitation of the polymer and continuous coating of the polymer on the core drug surface. The last step is the hardening or solidifying of the coated polymer materials on the core drug surface [240,241,242]. The mechanical process of drug entrapment in micro- or nanoparticulate systems includes fluid bed coating and the solvent evaporation technique [238]. In fluid bed coating, the drug particles are initially suspended in the airflow and then coated with a polymer solution through spraying. Subsequently, the solvent is evaporated to solidify the polymer coating around the drug particles [241,243]. However, the most widely used method for polymeric drug entrapment is the solvent evaporation or extraction method [244,245]. This method involves the emulsification of the polymer and drug solution, followed by the evaporation of the solvent. During the evaporation process, the polymer materials shrink around the core drug particles and produce micro- or nanosized particles [240,241]. The emulsification process of the polymer and drug solution depends on the physiochemical properties of the drug, which can be either a single emulsification process (o/w) for hydrophobic drugs or a double emulsification process (w/o/w) for hydrophilic drugs [241,246,247]. However, challenges still exist in using the above-mentioned processes for nano- or microparticle formulation as controlling size, size distribution, shape, and batch-to-batch variation remains unmanageable. Moreover, encapsulating biologics using these techniques is considerably more challenging due to the inherent nature of proteins and biologics to interact with the polymer surface, as well as concerns about the stability of the biologics [248,249]. 

#### 5.4.1. PLGA-Based Micro- and Nanoparticles

PLGA has ideal physiochemical properties as a drug carrier, such as biocompatibility and both gel- and capsule-forming ability [51,250]. The superior suitability of PLGA has been acknowledged in clinical settings, and the introduction of PLGA-based depot-forming injectables began with the US FDA’s approval of PLGA-based Lupron Depot in 1989. Since then, PLGA microparticles have been used for delivering both small molecules and protein, such as Nutropin Depot.

A lot of preclinical efforts were also employed to develop ARV-loaded PLGA micro- and nanoparticles, as the PLGA particle can encapsulate multiple ARVs in the same formulation. Those particles have shown promise to prolong dosage regimens for both treatment and pre-exposure prophylaxis (PrEP) as they improved the PK and drug release properties of the ARV. Tahir Khuroo et al. developed a PLGA microparticle encapsulating a prodrug of DTG by the emulsification–evaporation method. This PLGA microparticle increased the plasma apparent half-life of the DTG by almost 8.6-fold. However, the PLGA microparticle showed an initial burst release of DTG on day 1. However, the plasma DTG level gradually decreased by 4-fold over a 60-day period [251]. Initial burst release is a common phenomenon for PLGA-based formulations. Several studies on the morphology and drug distribution of PLGA microparticles indicated that PLGA microparticles are very porous and the drugs in the PLGA microparticles are distributed on the surface or the orifice of the large, interconnected pores [252,253]. Initially, water is absorbed through the pores, and then fast dissolution of the neighboring drugs may cause the initial burst release of the drugs. In an alternative scenario, water uptake into PLGA microspheres with a seemingly smooth, thin surface layer can directly initiate drug release through various processes [253]. These processes may involve polymer swelling, leading to the formation of surface pores or the creation of new pore networks. However, after the initial burst release, the PLGA microparticle often undergoes pore rearrangement or a process called pore healing, causing new pore formation and pore closing [253,254]. This may also explain the steady drug release kinetics after the initial burst release from PLGA microparticles. The drug release kinetics from PLGA-based micro- and nanoparticles can be controlled by controlling the MW of PLGA and the glycolic and lactic acid ratio of the PLGA and size and surface porosity of the particle [255,256,257].

PLGA nanoparticles have been widely employed by Mandal et al. and colleagues for delivering combination ARV (cARV), such as (EVG + TAF + FTC) or (TFV + EVG) or (TAF + FTC) or (BIC + TAF) [258,259,260,261]. These nanoparticles were developed by a w/o/w emulsion–solvent evaporation method, where the aqueous solution of the drugs was first emulsified with organic solution of PLGA (75:25 lactide:glycolide ratio) (MW 4000–15,000 Da) and poloxamer, then the resultant emulsion was again emulsified in 1% PVA solution to generate a nanoparticle with a size of 250 nm. The nanoparticle improved the biodistribution of all three encapsulated drugs. Four subsequent injections of these nanoparticles over a 6-week period protected humanized mice from HIV-1 infection for 19 weeks [261]. A similar nanoparticle encapsulating either TAF + FTC or TAF + BIC or TAF + EVG also improved the drug PK and biodistribution of those drugs [258,260,262].

Despite numerous clinically approved depot-forming PLGA-based implants and a significant increase in PLGA-based drug delivery development efforts over the last few decades, there are still notable limitations in PLGA-based drug delivery systems. Such limitations include the degradation of PLGA suspension or solution over the storage period due to its hydrolytic properties. To improve the efficiency and effectiveness of PLGA-based drug delivery, it is crucial to thoroughly understand the factors that influence the degradation rate and drug release kinetics of PLGA-based polymers [263]. In addition to that, injection site reaction, accumulation of immune cells, and deposition of micro- and nanoparticles in the spleen and lung can pose significant challenges for developing polymeric nano- and microparticles [264].

#### 5.4.2. Nanocrystal Formulations

Nanocrystal technology has revolutionized HIV-1 treatment and prevention. This technology facilitates formulation of water-insoluble molecules into LA surfactant-stabilized drug nanosuspensions in aqueous buffers. Using either top-down or bottom-up technology, such as high-pressure homogenization, wet bead milling, microfluidization, and the emulsion template freeze-drying technique, solid drug microparticles are suspended in an aqueous buffer with stabilizing surfactants or water-soluble polymers and nanoformulated into stable solid drug nanoparticles. Compared to other formulation approaches, nanocrystals exhibit superior properties that include high drug loading to affect administration volumes, improve drug dissolution through reduced particle size, protect drug molecules from rapid metabolism to extend their apparent half-lives, and enhance drug stability by keeping it in solid form within the formulation. The use of aqueous buffers to produce drug nanocrystals also minimizes solvent-related toxicities in the final product. The success of this technology in HIV therapy is highlighted by the LA injectable Cabenuva (CAB and RPV LA) and Apretude (CAB LA) which are currently approved for treatment and prevention, respectively (Table 1). LA CAB consists of pure CAB nanocrystals with an average particle size of 200 nm dispersed in a stabilizing aqueous buffer solution of Tween 20, PEG_3350_, and mannitol [265]. For LA RPV nanocrystals, Poloxamer 338 (P338) is used as the stabilizing surfactant [35].

Upon IM injection, these nanocrystals form a drug depot at the injection site that slowly dissociates to release the drug for months. Drug release and absorption in biological matrices are dependent on several factors such as nanoparticle agglomeration or dispersion at the injection site, particle size, and surface properties that affect host protein adsorption to the particle surface to facilitate absorption [265]. The longevity of the depot is heavily influenced by the hydrophobicity of the formulated compounds. However, to ensure sufficient dissolution of the nanocrystals into the surrounding medium and eventually demonstrate effective plasma and tissue drug levels, the hydrophobicity of the formulated compound needs to be optimal. The particle size, in addition to the hydrophobicity, also influences the dissolution properties. With a larger surface area, the nano-sized crystal improves the dissolution properties of the drugs, helping maintain a sufficient concentration for efficacy. The benefit of smaller particle size is also evident in the improved syringeability of the formulation [167,265].

To further extend ARV dosage regimens, a prodrug strategy that is coined as ‘long-acting slow effective antiretroviral therapy’ (LASER ART) was developed (Table 2). The strategy relies on controlled drug release from a tissue macrophage depot. LASER ART was applied to all classes of ARVs with the goal of improving their physicochemical properties to facilitate their formulation into scalable LA agents with improved apparent half-lives. Some are currently in advanced preclinical development with the potential to improve the HIV treatment dosage regimen by up to 6 months or longer [269].

Prodrug nanocrystals of a stearoylated CAB were shown to sustain CAB concentrations for up to one year in rodent and non-human primate (NHP) models [9]. A similar modification of DTG significantly improved the PK profile of the parent drug [269]. However, esterification of CAB and DTG with either shorter or longer than 18-carbon fatty acid failed to maintain plasma CAB and DTG levels beyond two months [269,270,271]. The prodrug nanocrystal strategy was extended to NRTIs where FTC, 3TC, and TFV were converted into hydrophobic ProTide prodrugs with improved PK profiles [272,273,274]. Notably, hydrophobic stable TFV ProTide nanocrystals demonstrated effective TFV-diphosphate levels in a two-month PK study in rats after a single IM injection [272]. This formulation demonstrated anti-HBV activity in both HBV-infected transgenic and human hepatocyte-transplanted TK-NOG mice, for at least three months [275,276].

**Figure 7:**
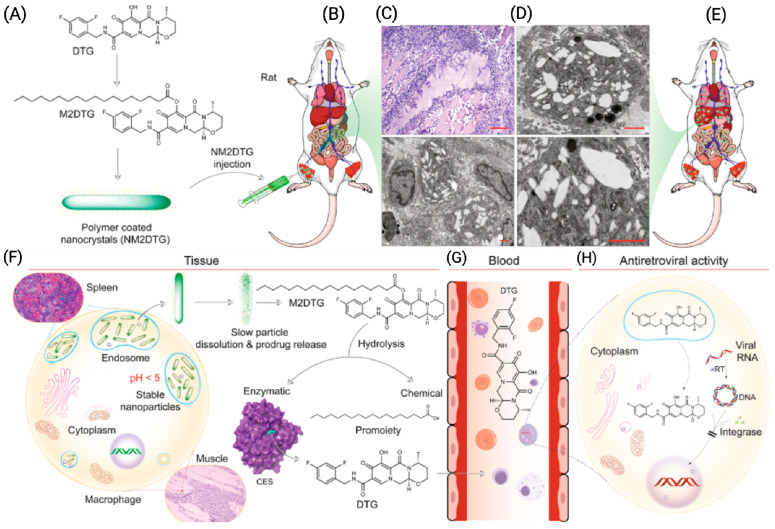
The illustration shows the in vivo fate of LASER ART prodrug nanoformulation. (**A**) Chemical synthesis of prodrug from parent ARV. (**B**) IM injection of LASER ART nanoformulation illustrates the muscle as the primary drug depot. (**C**,**D**) Macrophages phagocytose and store the nanocrystal from the site of injection. (**E**) Biodistribution of prodrug and active drug in tissues including HIV-1 reservoir site. (**F**) Slow prodrug release in the low-pH microenvironment in macrophages and then hydrolysis to release active drug. (**G**,**H**) Slow dissolution and hydrolysis of prodrug in the blood and tissues. The figure was reproduced under the terms of the Creative Commons CC-BY license [269].

Research across a broad spectrum of hydrophobic ARV prodrugs sheds light on the correlation between prodrug hydrophobicity and drug release. The prolonged PK profile observed in INSTI-esterified prodrugs is influenced by multiple factors that contribute to the observed extended apparent half-lives. These influencing factors encompass (i) the physiochemical attributes of the prodrug, (ii) the chemical and enzymatic conversion of the prodrug to the parent drug, and iii) the absorption of the prodrug and parent drug by the lymphatic systems and tissues (Figure 7). Yet, there is a noticeable gap in research dedicated to understanding the role of polymer/surfactants in drug release and how they interact with cells that undergo mononuclear phagocytosis. Many of these formulations require hydrophilic low-MW polymers like poloxamer or a combination of PEG and polysorbate. This ensures that the particles remain stable in aqueous suspensions. One crucial facet of nanocrystal formulations is their uptake by phagocytic cells, like macrophages. The nature of the polymer in the formulation affects how macrophages interact with the nanoparticles and how stable the nanoparticles remain within phagocytosed cells. Numerous studies highlight the stability of these nanoparticles within macrophages, pointing to the creation of a depot within these cells. Comprehensive research focusing on prodrug nanoparticle size, charge, polymer/surfactant choices, and their effects on macrophage interactions is crucial. Deodhar et al. demonstrate improved stability of surfactant-coated nanoformulated DTG prodrugs in various environments like acidic pH, macrophages, plasma, and various tissues, compared to their counterparts in a free prodrug solution [269]. Nevertheless, the foundational mechanism promoting the heightened stability of the nanoparticle calls for further exploration. Furthermore, various challenges are associated with this technology when the native parent is used in the formulation, including an initial burst release, a prolonged PK tail, and population-level PK variability [265,277]. This variability in CAB LA’s PK is also evident between genders. Some females exhibit a smoothed concentration–time curve, presenting a lower peak drug concentration (C_max_) and a prolonged period to reach this C_max_ compared to males. However, both genders show comparable overall drug exposure (AUC_[0–∞]_) following a single LA dose of CAB. This suggests that the absorption rate might differ between genders, rather than the total absorption [278]. Additionally, the terminal phase PK tail for CAB LA shows variations between male and female patients.

**Table 2:** LA ARV formulations that are either in clinical or preclinical development.

Delivery System	ARV Drug	Polymer	Formulation Details	Predicted Dosage Regimen (Month)	Clinical Trial	Ref.
Implant	TAF	Silicone and PVA	TAF powder is encapsulated in a silicone tube, which has several drug delivery channels coated with PVA. Drug release rate can be controlled by controlling the number of channels or thickness of PVA membrane.	6	Phase I	[15]
	TAF	Silicone	Transcutaneous refillable nanochannel delivery implant, where drug reservoir is made of titanium and drug is released through TaN-coated slit nanochannel.	6	Preclinical	[11]
	TAF	PUs	TAF formulation pellet is encased in PU-based drug reservoir and the drug is released through diffusion through the PU membrane.	3		[162]
	CAB		CAB formulation is converted into pellets and then encased in PU membrane containing drug reservoir. PU membrane acts as RCM for controlling drug release through diffusion.	3		[168]
	TAF	PCL	TAF formulation containing TAF and castor oil was loaded into PCL tubes and the drug was released through the diffusion and bulk erosion of PCL. Drug release rate can be controlled through controlling MW of PCL.	3		[160]
	ISL	EVA	Crystalline ISL is uniformly dispersed in EVA and the implant is prepared through hot melt extrusion.	12	On hold	[165]
ISFIs	CAB	PLGA	CAB and PLGA were solubilized in NMP:DMSO and injected subcutaneously. Upon injection, the solution went through phase inversion and formed solid implant.	6	Preclinical	[51]
	DTG		Same as CAB PLGA ISFI	6		[180]
VRs	TDF	PUs	TDF formulation was loaded in hydrophilic PU tubes and the PU tubes were end-sealed using induction welding.	1		[203]
	TDF	PUs	TDF and NaCl formulation was loaded onto PU extruded tubes and end-sealed to prepare the VRs.	1		[17]
MNs	CAB	PVP and PVA	MN tips were generated by using a hydrogel composed of 20:20:60 PVA:PVP:CAB and the baseplate of MNs was made using PVP and PVA. The MN tips dissolved quickly to release the CAB and the CAB formed drug depot at the administration site.	1	Preclinical	[22]
	BIC		MNs tips were made of PVP, PVA, and BIC. The baseplate was made of PVP and glycerol.	1		[230]
	RPV		MN tips were made of PVP and RPV. The baseplate was made of PVA and glycerol.	1		[23]
Microparticle	DTG	PLGA	DTG was transformed into a hydrophobic prodrug and encapsulated into PLGA-based microparticles by organic and aqueous solvent emulsification–evaporation.	3		[251]
Prodrug nanocrystal	TFV	Poloxamer, Polysorbate and PEG	TFV was converted into lipophilic ProTide and then formulated as aqueous nanocrystals.	≥3		[272]
	CAB		Lipohilic ester prodrug cabotegravir was synthesized and formulated as aqueous nanocrystals.	12		[9]
	DTG		Fatty acid ester prodrug DTG was synthesized and nanoformulated as aqueous nanocrystals.	≥6		[269]

ISL—islatravir, TFV—tenofovir, TDF—tenofovir disoproxil fumarate, TAF—tenofovir alafenamide fumarate, CAB—cabotegravir, BIC—bictegravir, DTG—dolutegravir, RPV—rilpivirine.

## 6. Conclusions and Future Perspective of LA Drug Delivery Systems

The prospects for polymer-based LA formulations are bright, given our rapidly expanding knowledge of polymer properties and drug release mechanisms. While current LA drugs for HIV offer improvement over traditional oral HIV medications, there are user- and treatment-related gaps that could be addressed by the emerging LA drug delivery technologies such as prodrug approaches and implants. Ideally, future LA ART medicines should possess three main characteristics.

First, they should be easy to manufacture, scalable, and user-friendly, with a dosing schedule extended at least beyond two months to synchronize dosing with the existing routine patient laboratory test clinic visits. A desirable target product profile is therefore a six-month ART dosage regimen. However, achieving a six-month dosage regimen with the exixting parent drugs is challenging due to their inherent features that include rapid drug metabolism upon absorption from the implant/injection site depot. The constraints on the allowable volume for injections or the size of implantable products, especially for SC or IM delivery, further intensify this challenge. Sometimes, the required drug amount surpasses these permissible limits. A potential solution lies in choosing drugs that are highly potent and have a high resistance to mutations. Prodrug approaches could also be used to achieve this goal by improving intracellular drug delivery and absorption, distribution, metabolism, and excretion (ADME) properties of parent drugs to extend their half-lives. When identifying the optimal drug for LA formulation development, coexisting medical conditions in patients should also be considered. Given that a significant number of HIV patients (~7.5%) are coinfected with hepatitis B or tuberculosis, LA drugs that can concurrently target multiple viruses are especially valuable [279].

The second desired feature of LA formulations is to minimize postmarket complications. This includes curtailing the prolonged PK tail in the terminal phase of drug release, inhibiting the emergence of drug-resistant mutations, and ensuring that the treatment can be reversed, especially for newer drugs with no established safety profiles, if adverse reactions occur. For example, the FDA-approved CAB LA exhibits a long PK tail after the last injection [280]. The CDC recommends that those at risk of HIV continue with oral CAB or PrEP for at least a year after discontinuing CAB LA. Yet, making the shift from LA drugs to oral PrEP is not always straightforward, as many prefer LA treatments over daily oral ART. There are also concerns about how easily LA treatments can be reversed in the event of complications. These have been circumvented through oral lead-ins prior to administering LA injectable agents. Solid LA implantable devices might offer an alterantive reversal solution, but the need for surgical removal postlifespan dilutes this advantage.

The third desired feature of LA formulations is their widespread adoption and utilization by the end users. Factors like cost, storage stability, transportation requirements, and specific storage conditions can significantly influence the acceptance and use of LA medicines. Given that HIV is most common in resource-limited settings with limited financial capacity for medication, inadequate cold storage infrastructure, and a scarcity of medical professionals, an LA medicine that is affordable, stable at room temperature, and self-administrable would greatly boost its adoption and application.

Currently, no LA technology, whether in clinical or preclinical development, embodies all three of these ideal features, suggesting that a ‘one size fits all’ approach to LA therapy is inappropriate. Given this, there is a push to tailor LA medicines to the unique needs of specific subgroups. Understanding the interactions between the polymers, drugs, and their release mechanisms will enable more precise tuning of drug release rates from these formulations. Additionally, to ensure the clinical success of LA ARVs in such resource-limited settings, it is imperative to involve all stakeholders in the early phase of formulation development. These stakeholders include end users, healthcare professionals, business developers, and policymakers. Collaboration, careful planning, and implementation consideration are essential to prioritize and design the most promising ARVs and LA DDSs that facilitate effective implementation in the most HIV-burdened settings. Moreover, the development of LA ARVs may present regulatory challenges, such as establishing optimal dosing regimens to address the need for combination antiretroviral therapy and managing missed doses and potential drug–drug interactions in patients with comorbidities. Some of these challenges are particularly important for PrEP dosing schedules, as there is no precise biomarker to gauge outcomes. In such situations, conducting thorough in vivo PK studies and evaluating PrEP effectiveness in preclinical animal models becomes imperative. Nonetheless, selecting an appropriate animal model to determine PrEP efficacy remains a significant challenge in preclinical evaluation, given that none of the existing models can fully replicate the human transmission and immunogenicity of HIV infection. Additionally, since LA ARVs can persist in the body for extended periods, ensuring the long-term safety of both the active ingredients and excipients used in LA ARVs in preclinical in vivo models is of significance. While the excipients used in LA ARVs are generally recognized as safe (GRAS), extensive GLP toxicology studies in relevant models are needed to broaden the usage in special populations such as pediatric patients. The requirement for extensive, extended safety study timelines introduces further challenges, including increased overall product development costs and time. In this context, the use of physiologically based pharmacokinetic (PBPK) modeling can be a valuable tool for predicting drug PK. This approach can also streamline formulation screening and expedite the development of LA products.

Overall, advancements in polymer chemistry and insights into their drug release mechanisms have paved the way for promising strategies to extend HIV-1 treatment dosage regimens beyond two months. Nevertheless, there are developmental and translational hurdles that must be overcome for these products to successfully transition to clinical application.

## Figures and Tables

**Table 1:** A comprehensive summary of CAB- and RPV-based LA delivery system for the treatment and prevention of HIV-1 infection.

**Drugs**	(CAB + RPV LA)	CAB LA
**Brand name**	Cabenuva.	Apretude or Vocabria.
**Formulation characteristics**	CAB nanocrystals are produced in an aqueous solution of polysorbate 20 (Tween 20), PEG_3350_, and mannitol with particle size of 200 nm. RPV nanocrystals are produced in an aqueous solution of Poloxamer 338 with an average particle size of 200 nm.	Same as Cabenuva.
**Indication**	Treatment of HIV-1 infection.	Prevention of HIV-1 infection
**Population**	Virologically suppressed PLWH.	Adults and adolescents weighing at least 35 kg (77 lbs) who are at risk of sexually acquiring HIV.
**Dosage regimen**	Two initial injections of CAB 600 mg/3 mL and RPV 900 mg/3 mL given 1 month apart for two consecutive months and then given every two months (CAB 600 mg/3 mL and 900 mg/3 mL) thereafter. Injection is given in separate gluteal muscles.	Two initial injections (600 mg; 3 mL) given one month apart for two consecutive months, followed by maintenance doses (600 mg; 3 mL) given every 2 months thereafter. An OLI of CAB tablets may, or may not, be given for one month prior to starting CAB to assess tolerability.
**Approval**	First approved by Health Canada in March 2020, followed by the EMA in October 2020 and the US FDA in January 2021.	First approved by the US FDA in December 2021 and the EMA in October 2022.
**Efficacy in the clinical trials**	Several phase IIb and phase III clinical trials (LATTE-2, FLAIR, ATLAS, ATLAS-2M) proved non-inferiority for maintaining viral suppression compared to standard daily oral therapy [3,6,8,266].	Proved superior to approved oral PrEP agent TDF/FTC in 2 phase III clinical trials (HPTN-083 and 084) [267,268].
**Side effects**	Mild ISRs were the most reported AEs during clinical trials, but none of them caused treatment withdrawal.	Same as Cabenuva.

PLWH—people living with HIV. EMA—European Medicines Agency. US FDA—US Food and Drug Administration. OLI—oral lead in. AEs—adverse events. ISRs—injection site reactions.

## Data Availability

Data sharing is not applicable. No new data were created in this study.
